# Randomized Controlled Trial of Parent Therapeutic Education on Antibiotics to Improve Parent Satisfaction and Attitudes in a Pediatric Emergency Department

**DOI:** 10.1371/journal.pone.0075590

**Published:** 2013-09-26

**Authors:** François Angoulvant, Anne Rouault, Sonia Prot-Labarthe, Priscilla Boizeau, David Skurnik, Laurence Morin, Jean-Christophe Mercier, Corinne Alberti, Olivier Bourdon

**Affiliations:** 1 AP-HP, Hôpital Robert Debré, Université Paris Diderot, Sorbonne Paris Cité, Service d’Accueil des Urgences Pédiatriques, Paris, France; 2 AP-HP, Hôpital Robert Debré, Université Paris Diderot, Sorbonne Paris Cité, Unité d’Épidémiologie Clinique INSERM CIE5, Paris, France; 3 AP-HP, Hôpital Robert Debré, Faculté de Pharmacie, Université Paris Descartes, Département de Pharmacie, Paris, France; 4 Université Paris 13-Bobigny, Sorbonne Paris Cité. Laboratoire de Pédagogie de la Santé EA 3412, Paris, France; 5 Division of Infectious Diseases, Department of Medicine, Brigham and Women’s Hospital and Harvard Medical School, Boston, MA, USA; Columbia University, United States of America

## Abstract

**Objective:**

To evaluate therapeutic education delivered in a pediatric emergency department to improve parents’ satisfaction and attitudes about judicious antibiotic use.

**Methods:**

In an emergency department of a tertiary pediatric hospital, children aged 1 month to 6 years and discharged with an oral antibiotic prescription for an acute respiratory or urinary tract infection were randomized to a patient therapeutic education on antibiotic use (intervention group) or fever control (control group) delivered to the parents (in the presence of the children) by a pharmacist trained in therapeutic education. Education consisted in a 30-minute face-to-face session with four components: educational diagnosis, educational contract, education, and evaluation. The main outcome measure was parent satisfaction about information on antibiotics received at the hospital, as assessed by a telephone interview on day 14. The secondary outcome was attitudes about antibiotic use evaluated on day 14 and at month 6.

**Results:**

Of the 300 randomized children, 150 per arm, 259 were evaluated on day 14. Parent satisfaction with information on antibiotics was higher in the intervention group (125/129, 96.9%, versus 108/130, 83.0%; *P*=0.002, exact Fisher test).

**Intervention:**

Group parents had higher proportions of correct answers on day 14 to questions on attitudes about judicious antibiotic use than did control-group parents (*P*=0.017, Mann-Whitney U test).

**Conclusion:**

Therapeutic education delivered by a clinical pharmacist in the pediatric emergency department holds promise for improving the use of antibiotics prescribed to pediatric outpatients.

**Trial Registration:**

ClinicalTrials.gov NCT00948779 http://clinicaltrials.gov/show/NCT00948779

## Introduction

Infections caused by antibiotic-resistant microorganisms are associated with high morbidity, mortality, and healthcare costs [1]. Antibiotic overuse promotes the emergence of drug-resistant bacterial strains [2-4]. Equally deleterious is antibiotic misuse, which may consist in failure to complete the prescribed course; skipped doses; or use of antibiotics left over from a previous illness, resulting in the intake of an inadequate dose without prior evaluation by a physician [5,6]. Such behaviors have been reported to increase antibiotic resistance rates, treatment failure rates, and costs [6].

Acute respiratory tract infections (ARTIs) are common reasons for antibiotic prescription [7]. The number of unnecessary antibiotic prescriptions for ARTIs was estimated at 22.6 million in 1998 in the US, corresponding to a cost of about $726 million [8]. In 2010 in France, 10.4 million antibiotic prescriptions were given to children, mainly for ARTIs [9]. In children, several studies in Europe and the US established that ARTIs were the main reason for antibiotic prescription in both private offices and pediatric emergency departments (PEDs) [7,10-13]. In 2006 in the US, these infections were the reason for 78% of antibiotic prescriptions in children younger than 5 years [7].

Decreasing antibiotic overuse and misuse is a crucial objective in the fight against antibiotic resistance [4,14]. International guidelines recommend multiple interventions including education for healthcare professionals and the public about the rational use of antibiotics [15,16]. National campaigns to educate the public may improve antibiotic use [17], but individually customized interventions delivered to patients and to the parents of pediatric patients may be required also [16]. The expectations and behaviors of parents can heavily influence physicians’ attitudes concerning antibiotic prescription [10,18], leading to antibiotic overuse in private practice and PEDs, whereas poor physician-patient rapport in primary care is associated with failure to fill prescriptions [19]. Good understanding of the treatment by the parents may significantly improve treatment adherence [18-22]. Greater parent satisfaction with pediatric care correlated with improved quality of care in the child via better comprehension of medical information and increased treatment adherence [21,23-25]. Thus, in addition to being a major healthcare objective, satisfaction is associated with significant improvements in several important outcomes [23-26].

Visits to the PED provide an opportunity for health-promotion and family-education interventions [20,27]. To our knowledge, no studies on active therapeutic education about appropriate antibiotic use delivered to parents in PEDs have been published to date. Therapeutic education is used chiefly for patients with chronic diseases [28], but interactive educational interventions delivered to patients by healthcare professionals may improve the knowledge and skills needed to optimize antibiotic use [16].

We evaluated a therapeutic education intervention designed to improve skills useful for a better adherence to prescribed antibiotics and delivered by clinical pharmacists to parents and children seen in a PED in Paris, France. We then conducted a randomized controlled trial to assess the effect of this intervention on parent satisfaction and attitudes about antibiotic use in children prescribed antibiotics for an ARTI or urinary tract infection.

## Methods

### Study design and setting

The protocol for this trial and supporting CONSORT checklist are available as supporting information; see Checklist S1 and Protocol S1. This randomized controlled trial with two parallel groups was conducted in the PED of the Robert Debré Hospital (Assistance Publique-Hôpitaux de Paris), Paris, France, a mother-child teaching hospital serving a culturally and linguistically diverse population.

### Ethics

This study was approved by the appropriate biomedical research ethics committee in February 2009 (Bichat-Claude Bernard Hospital, Paris, France; #IRB0006477; n° 08-071). No incentives to participation were offered. Oral informed consent was obtained from at least one of the parents or legal guardian; hereafter, the term “parents” is used for both. The study was registered on Clinicaltrial.gov (NCT00948779) on July 28, 2009. The education sessions were delivered to the parents accompanying the children.

### Selection of participants

Patients meeting study criteria were enrolled from Monday to Friday between 9 a.m. and 6 p.m., from February 2, 2009 to September 26, 2011.

Children between 1 month and 6 years of age were eligible if they were discharged from the PED with a prescription for 5 to 10 days of an oral liquid antibiotic to treat an ARTI (mainly tonsillitis, acute otitis media, lower respiratory tract infection, or adenitis) or an urinary tract infection (pyelonephritis or cystitis). The criteria regarding diagnoses and treatment duration were chosen to produce a homogeneous population. No specific instructions were given to the physicians, who delivered the usual information about fever control and antibiotic treatment to the parents. Exclusion criteria were chronic conditions affecting drug dosing, suspected or known allergy to the prescribed antibiotics, and no telephone number for contacting the parents. No patient could be included more than once. Parents having poor knowledge of French were excluded because we did not have the resources needed to deliver education sessions in other languages. The parents of eligible patients were informed about the study orally and via a printed information sheet. To ensure blinding of the parents, the existence of two groups was not disclosed. Once informed consent was obtained, patients were enrolled by the PED physicians and referred to a clinical pharmacist on site. The children were allocated at random in a 1:1 ratio to therapeutic education on antibiotic use (the intervention group) or on fever control (the control group). A block randomization scheme with variable block length was previously generated using a computer. To ensure concealment, the clinical pharmacist used an Intranet connection to obtain the group assignment of each included patient.

### Interventions

The intervention and control sessions were designed by a clinical pharmacist trained in therapeutic education, in compliance with 1998 World Health Organization (WHO) and 2007 French National Authority for Health (HAS) recommendations [28,29]. Five clinical pharmacists trained in therapeutic education delivered the sessions. The 30-minute face-to-face sessions with the parents of each patient were held in a private room. When both the mother and the father accompanied the child, both attended the educational session.

The contents of the intervention and control sessions were similar in terms of the teaching tools and methods but differed regarding the topic discussed, which was antibiotic use or fever control. The teaching goals for the families in the intervention group were as follows: good understanding of antibiotic therapy effects on the diagnosed disease, good understanding of treatment modalities, correct preparation and administration of the antibiotic (preventing spitting, masking the flavor…), awareness of potential undesirable effects and ability to manage these effects, ability to explain the importance of the antibiotic treatment to all the child’s caregivers, familiarity with the concept of generic antibiotics, and ability to specify to the pharmacist filling the prescription which flavor is desirable. The control session had the following teaching goals: good understanding of the pharmacological and physical means available for controlling fever, ability to use these means appropriately, ability to administer antipyretic medications to the child, and ability to explain the importance and modalities of pharmacological and physical fever-control measures to all the child’s caregivers.

The pharmacists delivering the education used an interview grid to ensure standardization of the sessions. As the intention-to-treat approach was used, they could answer all the parents’ questions, even those on fever control in the intervention group and those on antibiotics in the control group. Sessions were organized according to the four stages recommended by the WHO: educational diagnosis, educational contract, education, and evaluation [28]. The educator acted as a resource and guide for the discussion: interactivity was encouraged. Drawing tools such as Barrows cards were created to support the sessions [30], and illustrated information sheets were given to the parents at the end of the session. Each session covered five topics: (1) identification of knowledge, beliefs, and behaviors concerning prescribed treatments and comprehension of their effects; (2) mutual determination of objectives to be achieved by the session; (3) education about oral liquid preparation, administration, storage, and possible side effects; (4) maximizing adherence in everyday situations (Barrows cards exercise) and identifying the best dosing time during the day to minimize the risk of skipped doses; and (5) brief assessment of comprehension by the parents via an open question on what should be done to ensure effectiveness of the prescribed treatment. Table **1** shows the main parts of the two sessions, both of which combined the three components (educational, behavioral, and organizational) in the classification developed by Wu et al. in 2008 [31].

**Table 1:** Conduct of the educational sessions.

Intervention session	Control session	Teaching tools
(1) Presentation of the session		
(2) Identification of knowledge, beliefs, and behaviors concerning prescribed treatments and prescription understanding in regard to the disease		Interactive discussion with well-defined opening questions about antibiotics or fever and their management
(3) Mutual determination of objectives to be achieved by the session		Interactive discussion
(4) Education about oral liquid preparation, administration, storage or side effects likely to arise; solutions to avoid spits, mask flavors…		Practice demonstration with different forms of oral solutions of the prescribed antibiotics/antipyretics ; Discussion around drawings and illustrated information sheets about the prescribed antibiotics/antipyretics
(5) Maximizing adherence in everyday situations: role-playing; identifying the best dosing time during the day to minimize the risk of skipped doses	(5) Improve fever control in everyday situations: role-playing; knowledge of fever-control measures; antipyretic regimen; physical treatments; when to see a doctor…	Barrows cards exercise about adherence to antibiotic therapy or fever control; Discussion around drawings and illustrated information sheets about the antibiotics/fever control
(6) Brief assessment of comprehension by the parents		Open question on what should be done to ensure the effectiveness of the prescribed antibiotics/fever control measures

### Measurement methods

The impact of the education sessions was assessed by administering a questionnaire over the telephone 14 days and 6 months after the PED visit. Pharmacists blinded to group allocation and trained to administer the questionnaire conducted the assessments. For the first assessment, at least three attempts to contact each family were made between day 14 and day 17 inclusive. The questionnaire consisted of 11 standardized structured questions on satisfaction and attitudes (Table **2**). The items on attitudes consisted chiefly in questions about judicious antibiotic use that were derived from the questionnaire developed by Pechere et al. [32] and evaluated a combination of knowledge and practice. Replies were given using a 1-to-5 Likert scale. The response options for the satisfaction scale were 1, very dissatisfied; 2, mostly dissatisfied; 3, neither satisfied nor dissatisfied; 4, mostly satisfied; and 5, very satisfied. For the agreement scale, response options were 1, strongly disagree; 2, mostly disagree; 3, neither agree nor disagree; 4, mostly agree; and 5, strongly agree. Finally, options for the difficulty scale were 1, very difficult; 2, rather difficult; 3, rather easy; and 4, very easy. The second assessment was performed 5 to 7 months after the PED visit and served only to evaluate attitudes concerning antibiotic use [32]. Questionnaire reliability was checked in 10 individuals before study initiation.

**Table 2:** Telephone questionnaire used for the first assessment on day 14.

***1.***	**Has the infection resolved? ❒ yes ❒ no; If not, was another antibiotic prescribed to your child? ❒ yes ❒ no; What is the name of the antibiotic prescribed? |__|**
***2.***	***How satisfied are you about the information on antibiotics received at the hospital: |__| (Satisfaction scale)***
***3.***	***How satisfied are you about the information on fever control received at the hospital?: |__| (Satisfaction scale)***
***4.***	***How difficult was it to have your child take the antibiotic solution on schedule? |__| (Difficulty scale)***
***5.***	***To what extent do you agree with the following statement: If my child is healed /cured/ feeling better, I sometimes save the rest of the antibiotics for the next time he get sick |__| (Agreement scale)***
***6.***	***To what extent do you agree with the following statement: Antibiotics are only effective if my child finishes all of them, even if my child*’s *symptoms are already gone: |__|* (*Agreement scale*)**
***7.***	***To what extent do you agree with the following statement: I always follow the doctor’s instructions exactly when my child is taking an antibiotic: |__| (Agreement scale)***
***8.***	***To what extent do you agree with the following statement: Left over antibiotics can be saved and used again: |__| (Agreement scale)***
***9.***	***To what extent do you agree with the following statement: If my child doesn’t finish all of the antibiotics, some of the germs may survive: |__| (Agreement scale)***
***10.***	***To what extent do you agree with the following statement: Taking a few antibiotic doses is better than taking none at all: |__| (Agreement scale)***
***11.***	***Have you any comments? For example, did you have any trouble administering the antibiotic? Did your child dislike the taste of the antibiotic? Something else to say? …***

### Data collection and processing

For each patient, we recorded demographic data, the current diagnosis, name(s) of prescribed antibiotics, dose (mg/kg/day) and duration prescribed, and best telephone number for subsequently contacting the parents. If possible, two phone numbers were collected. Use of the best telephone number has been shown to improve contact rates compared to use of the number in the medical record [33,34].

### Outcome measures

The primary outcome measure was the percentage of parents satisfied with information on antibiotic therapy received in the PED, during the physician visit and the educational session [19,23-25]. The secondary endpoints were parent satisfaction about information received in the PED about fever control, parent attitudes about judicious antibiotic use (6 of the 11 questionnaire items on day 14) [32], and outcome of the acute infection.

### Primary data analysis

At least 136 patients were required in each group to detect a 15% difference in the proportions of satisfied parents between the two groups (80% versus 65%) with 80% power and a two-tailed α value of 0.05. The value in the control group was estimated from the literature and the value in the intervention group was the smallest improvement deemed relevant [18]. We did not compute the sample size needed to detect a significant difference regarding attitudes. We expected to be able to contact 90% of the families and we consequently planned to include 150 patients in each arm.

Statistical analyses were performed using SAS^®^ (v9.12, SAS Institute Inc., Cary, NC, USA). Qualitative variables were described as frequencies (percentages) and quantitative variables as means (SD) when distribution was normal and as medians (quartiles 1 and 3) otherwise. The Likert-scale replies on satisfaction were dichotomized as dissatisfied or satisfied with the neutral response classified as dissatisfied [19]; similarly, replies on attitudes were dichotomized as correct and incorrect [32]. For questions 5, 8, and 10 in Table **2**, “not at all agree” or “mostly disagree” replies were classified as correct and other replies as incorrect, whereas for questions 6, 7, and 9 in Table **2**, the correct replies were “mostly agree” or “strongly agree”. The two groups were compared using the intent-to-treat approach and either parametric or nonparametric tests depending on the nature and distribution of the variables. Qualitative measures were compared using the chi-square test or exact Fisher test depending on variable distribution. For between-group comparisons of the number of correct answers to attitude questions on day 14 and at month 6, we used the Mann Whitney U test. A Poisson Generalized Estimating Equations model was used to estimate changes in the number of correct answers between the first and second assessments in both groups, to take the non-independence of the data into account. To look for potential bias, we performed an additional analysis comparing day-14 data in the two groups depending on whether a single parent or both parents attended the educational session. Two-tailed *P* values of 0.05 or less were considered significant. No interim analyses were planned or performed.

## Results

### Characteristics of the study population

**Figure 1:**
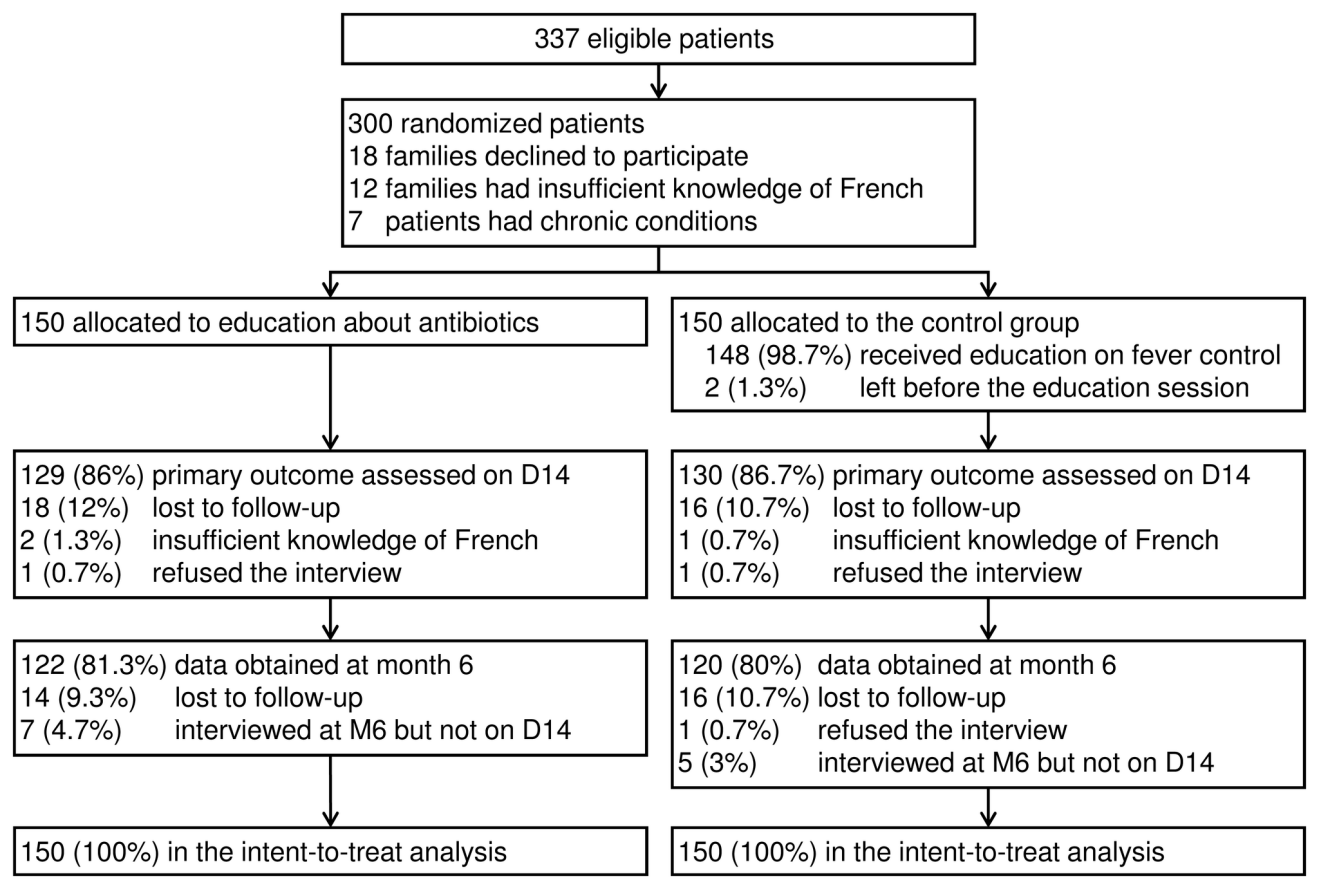
Participant flow chart.

Figure **1** is the participant flow chart. Table **3** reports the main baseline characteristics. We included 300 patients from February 2009 to September 2011, and all assessments were completed by April 2012. Of the 300 families, 2 (0.7%) left the PED before the education session and 298 (99.3%) participated in the full session (Figure **1**). During the education session, 5 families in the intervention group asked questions about fever control and 6 families in the control group asked questions about antibiotics.

**Table 3:** Baseline characteristics of patients overall and in each randomly assigned group.

	**Total N=300**		**Intervention group N=150**	**Control group N=150**	**Missing data**		***P***
**Female sex**					N=0		
N (%)	150 (50.0%)		76 (50.7%)	74 (49.3%)			0.8^a^
**Age in months**					N=0		
Median (Q1; Q3)	19 (11;36)		18 (10;33)	20 (11;37)			0.3^b^
**Weight in kg**					N=1		
Median (Q1; Q3)	11.6 (9.2;15.0)		11.3 (8.8;14.5)	12.0 (9.4;15.0)			
**Country of birth**					N=2		0.4^cd^
France	289/298 (97.0%)		145/150 (96.7%)	144/148 (97.3%)			
Algeria	3/298 (1.0%)		2/150 (1.3%)	1/148 (0.7%)			
Tunisia	2/298 (0.7%)		0/150 (0.0%)	2/148 (1.4%)			
Belgium	1/298 (0.3%)		1/150 (0.7%)	0/148 (0.0%)			
Italy	1/298 (0.3%)		1/150 (0.7%)	0/148 (0.0%)			
Nigeria	1/298 (0.3%)		1/150 (0.7%)	0/148 (0.0%)			
Senegal	1/298 (0.3%)		0/150 (0.0%)	1/148 (0.7%)			
**Parents born in France**							
Father	121/294 (41.2%)		64/150 (42.7%)	57/144 (39.6%)	N=6		0.6^a^
Mother	129/296 (43.6%)		66/150 (44.0%)	63/146 (43.2%)	N=4		0.9^a^
**Accompanying adult(s)**					N=1		0.3^c^
Mother	206/299 (68.9%)		97/150 (64.7%)	109/149 (73.2%)			
Father and mother	63/299 (21.1%)		36/150 (24.0%)	27/149 (18.1%)			
Father	29/299 (9.7%)		17/150 (11.3%)	12/149 (8.1%)			
Legal guardian	1/299 (0.3%)		0/150 (0.0%)	1/149 (0.7%)			
		**Total N=300**	**Intervention group N=150**	**Control group N=150**	**Missing data**	***P***		
**Diagnosis**					N=1	0.6^c^		
Acute otitis media		107/300 (35.8%)	49/150 (32.7%)	58/149 (38.9%)				
Urinary tract infection		82/300 (27.4%)	41/150 (27.3%)	41/149 (27.5%)				
Lower respiratory tract infection		75/300 (25.1%)	41/150 (27.3%)	34/149 (22.8%)				
Tonsillitis		27/300 (9.0%)	14/150 (9.3%)	13/149 (8.7%)				
Adenitis		5/300 (1.7%)	3/150 (2.0%)	2/149 (1.3%)				
Other		3/300 (1.0%)	2/150 (1.3%)	1/149 (0.7%)				
**Antibiotic prescribed**					N=2	0.6^c^		
Amoxicillin + clavulanate		120/298 (40.3%)	61 (40.7%)	59 (39.9%)				
Amoxicillin		77/298 (25.8%)	42 (28.0%)	35 (23.7%)				
Azithromycin		1/298 (0.3%)	0 (0%)	1 (0.7%)				
Cefixime		80/298 (26.8%)	39 (26.0%)	41 (27.7%)				
Cefpodoxime proxetil		17/298 (5.7%)	7 (4.7%)	10 (6.7%)				
Cotrimoxazole		1/298 (0.3%)	1 (0.7%)	0 (0%)				
Josamycin		2/298 (0.7%)	0 (0%)	2 (1.4%)				

^a^ chi-square test, ^b^ Mann Whitney U test, ^c^ exact Fisher test ^d^ comparison of children born in France versus other countries.

Acute otitis media was the leading reason for antibiotic prescription and amoxicillin+clavulanate was the most often prescribed antibiotic (Table **3**). In 7 cases (2.3%), two different antibiotics were prescribed. Median prescribed treatment duration was 10 days and median prescribed number of doses was 3 per day.

### First assessment (day 14)

The proportion of parents satisfied with the information on antibiotics received in the PED (primary outcome measure) was significantly higher in the intervention group than in the control group (96.9% versus 83%, *P*=0.002) (Table **4**).

**Table 4:** Results of the first assessment on day 14.

**Questions**	**Correct answers**	**Intervention group %(n=129)^a^**	**Control group %(n=130)^a^**	***P* value**
How satisfied are you about the information on antibiotics received at the hospital?	Satisfied	96.9% (125)	83.0% (108)	0.002^b^
How satisfied are you about the information on fever control received at the hospital?	Satisfied	89.1% (115)	96.9% (126)	0.01^b^
How difficult was it to have your child take the antibiotic solution on schedule?	Easy	72.1% (93)	74.6% (97)	0.65^c^
Has the infection resolved?	Cured	86.8% (112)	93.1% (121)	0.09^c^
What is the name of the antibiotic prescribed ?	Known	76.7% (99)	70.3% (86)	0.06^c^

^a^ percentages of correct answers; ^b^ exact Fisher test ^c^ chi-square test

Among secondary outcome measures, the proportion of parents satisfied with information on fever control was significantly higher in the control group, where the session dealt with this topic, than in the intervention group.

**Figure 2:**
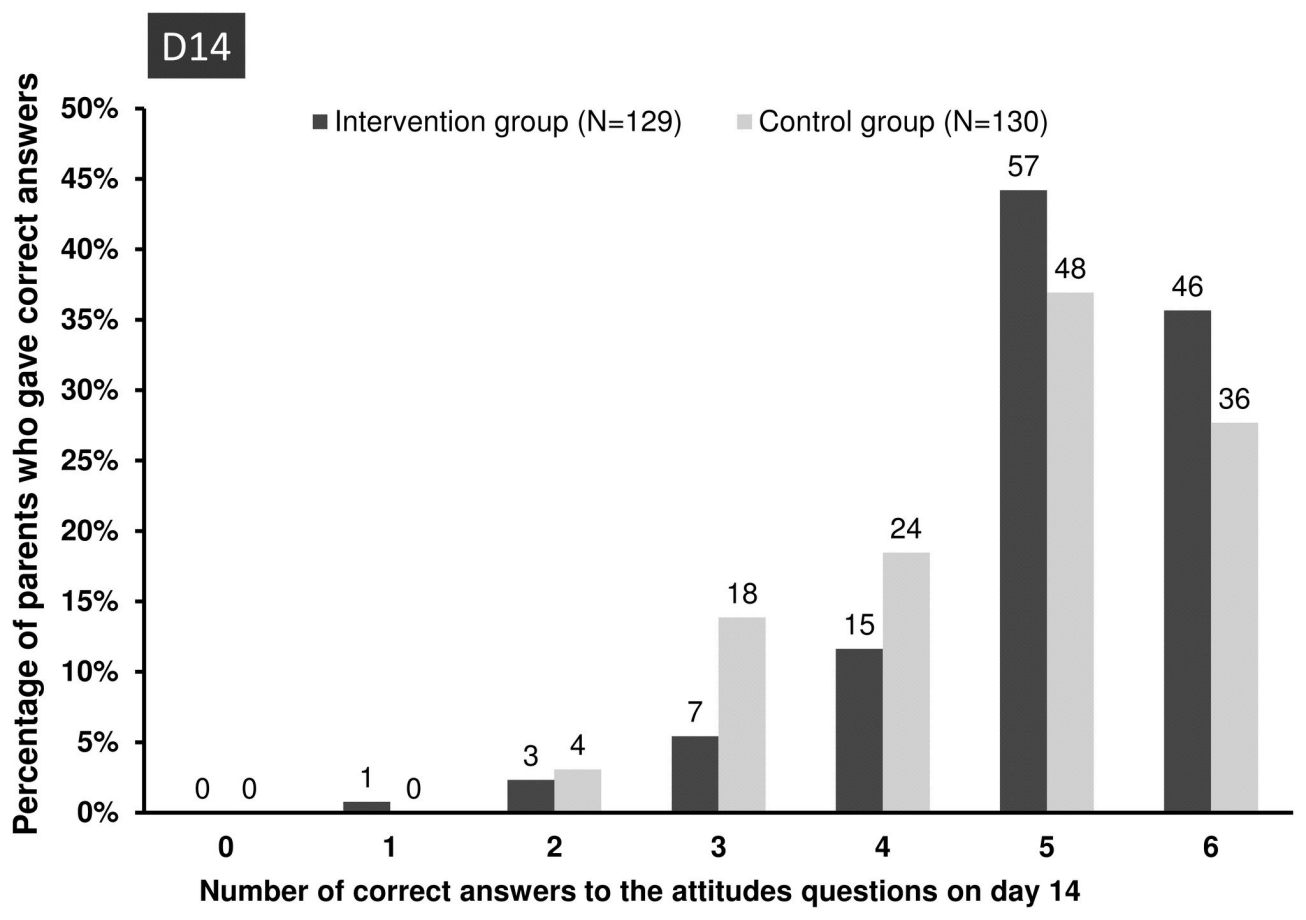
Percentage of correct answers about judicious antibiotic use at the first assessment on day 14. The numbers on top of the bars are the absolute numbers of parents who gave correct answers on day 14.

Parents in the intervention group gave significantly higher numbers of correct answers on day 14 to the questions on attitudes about judicious antibiotic use than did parents in the control group (*P*=0.017, Mann-Whitney U test). The proportions of parents who gave correct answers to 5 and 6 of these questions were 44% (57/129) and 36% (46/129), respectively, in the intervention group and 37% (48/130) and 28% (36/130), respectively, in the control group (Figure **2**). This difference in attitudes between the two groups was chiefly ascribable to two questions: “*Leftover antibiotics can be saved for later use*” (*P*=0.04) and “If my child does not take all the antibiotic prescribed, some of the germs may survive” (*P*=0.02) (Table **5**). No difference was found in the proportion of correct answers to the question “Taking a few antibiotic doses is better than taking none at all”, which had the lowest proportion of correct answers (about 50%) in both groups.

**Table 5:** Results of the questions on attitudes at the first assessment on day 14.

**Questions**	**Correct answers**	**Intervention group %(n=129)^a^**	**Control group %(n=130)^a^**	***P* value**
“If my child is feeling better, I sometimes save the rest of the antibiotic for the next time he get sick.”	Disagree	91.5% (118)	90.0% (117)	0.68^c^
“Antibiotics are only effective if my child finishes all of them, even if my child’s symptoms are already gone.”	Agree	91.5% (118)	87.7% (114)	0.32^c^
“I always follow the doctor’s instructions exactly when my child is taking an antibiotic.”	Agree	98.4% (127)	97.7% (127)	0.66^b^
“Left over antibiotics can be saved and used again.”	Disagree	77.5% (100)	66.2% (86)	0.04^c^
“If my child doesn’t finish all of the antibiotic, some of the germs may survive.”	Agree	92.2% (119)	83.0% (108)	0.02^c^
“Taking a few antibiotic doses is better than taking none at all.”	Disagree	51.9% (67)	48.5% (63)	0.58^c^

^a^ percentages of correct answers, ^b^ exact Fisher test, ^c^ chi-square test

Because the number of missing data was greater than 10%, we performed an additional analysis to ensure compliance with the intent-to-treat approach [35]. Missing data were replaced by unfavorable results (dissatisfaction for the primary outcome). The proportion of parents satisfied with the information on antibiotics remained significantly higher in the intervention group (124/150 [82.7%] versus 108/150 [72%]; *P*=0.018. Baseline characteristics did not differ between intervention and control group (Table **3**) and neither between the group lost to follow-up (N=38) and the group with complete data on day 14 (N=259) (Table **6**).

**Table 6:** Baseline characteristics in the group lost to follow-up and in the group evaluated on day 14.

	**lost to follow-up N=38**	**evaluated on D14 -N=259**	**Missing data**	***P* value**
**Female sex**			N=0	
N (%)	53% (20)	49% (128)		0.7^a^
**Age in months**			N=0	
Median (Q1; Q3)	25 (12;42)	18 (10;33)		0.2^b^
**Parents born in France**				
Father	39% (15)	42% (106)	N=4	0.8^a^
Mother	42% (16)	44% (113)	N=2	0.8^a^
**Accompanying adult(s)**			N=0	0.5^c^
Mother	76% (29)	68% (175)		
Father and mother	13% (5)	22% (58)		
Father	11% (4)	10% (25)		
Legal guardian	0% (0)	0.4% (1)		
**Diagnosis**			N=0	0.5^c^
Acute otitis media	42% (16)	35% (91)		
Urinary tract infection	21% (8)	29% (74)		
Lower respiratory tract infection	21% (8)	26% (67)		
Tonsillitis	16% (6)	8% (21)		
Adenitis	0% (0)	2% (4)		
Other	0% (0)	1% (2)		
**Antibiotic prescribed**			N=0	0.5^c^
Amoxicillin + clavulanate	45% (17)	39% (102)		
Amoxicillin	29% (11)	25% (66)		
Azithromycin	0% (0)	0.4% (1)		
Cefixime	21% (8)	28% (72)		
Cefpodoxime proxetil	3% (1)	6% (16)		
Cotrimoxazole	0% (0)	0.4% (1)		
Josamycin	3% (1)	0.4% (1)		

^a^ chi-square test, ^b^ Mann Whitney U test, ^c^ exact Fisher test

The number of phone calls made for the first assessment ranged from 1 to 9. The median time needed to administer the questionnaire over the telephone was 10 minutes. The mothers completed the first assessment in 103 (79.8%) cases in the intervention group versus 101 (77.7%) cases in the control group (*P*=0.67, Pearson’s chi-square test). The parent who completed the first assessment had attended the education session in 232 (89.6%) cases. We found no difference between the subgroups with one versus both parents having attended the educational session in terms of satisfaction with information about antibiotic use (90.1% [181] versus 89.7% [52], *P*=0.93, exact Fisher test) or about fever control (94.0% [189] versus 89.7% [52], *P*=0.25). Neither did we find any significant difference in the number of correct answers to the questions on attitudes about judicious antibiotic use (*P*=0.07, Mann-Whitney U test). For one family, the first assessment was conducted during a face-to-face interview because the child was admitted to the PED 14 days after the first visit.

### Second assessment (month 6)

**Figure 3:**
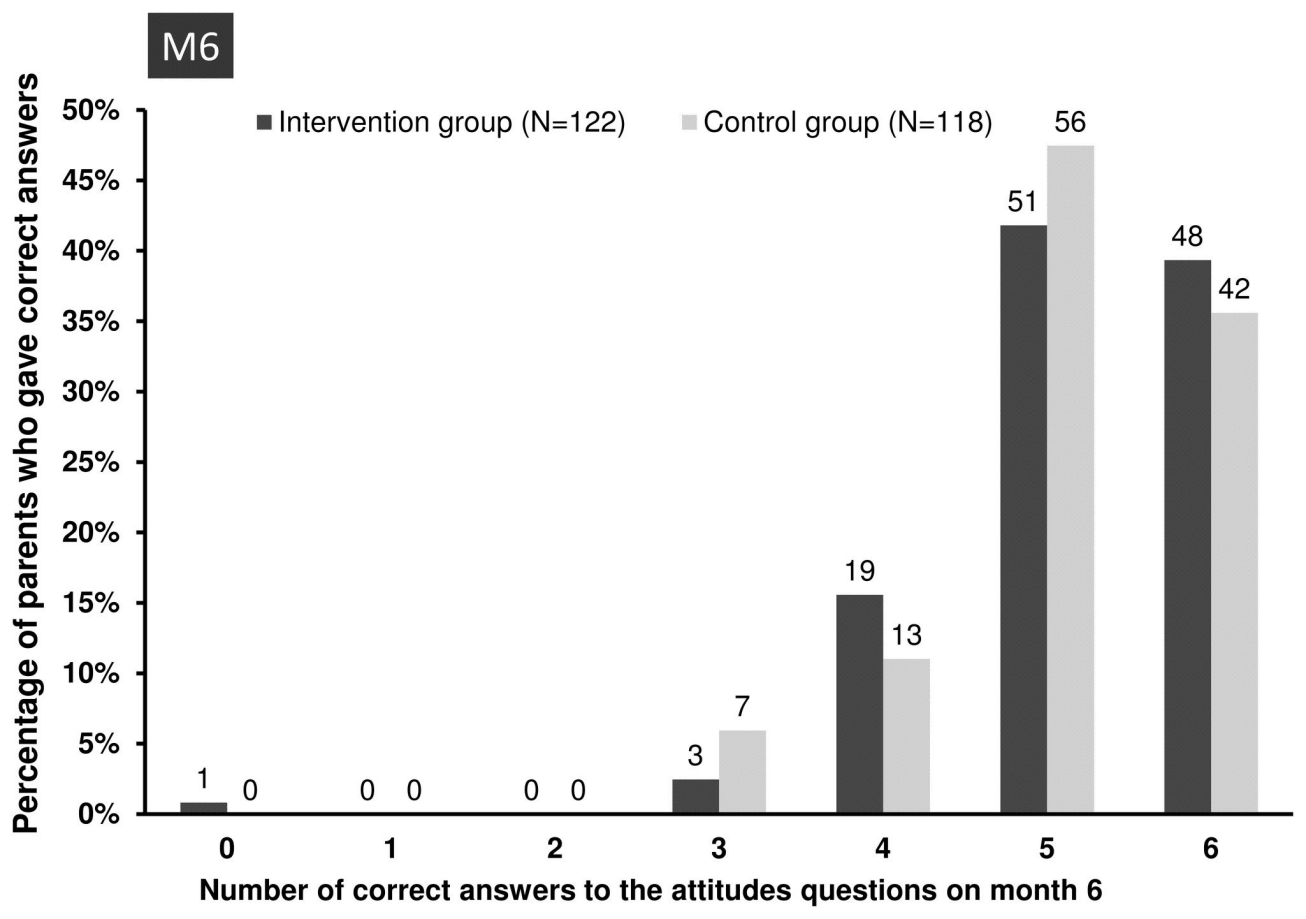
Percentage of correct answers about judicious antibiotic use at the second assessment at month 6. The numbers on top of the bars are the absolute numbers of parents who gave correct answers at month 6.

After 6 months, the proportions of parents who gave correct answers to questions on attitudes about judicious antibiotic use were not significantly different in the two groups (*P*=0.73, Mann-Whitney U test). The proportions of parents who gave correct answers to 5 and 6 questions on attitudes were 42% (51/122) and 39% (48/122), respectively, in the intervention group, and 47% (56/118) and 36% (42/118), respectively, in the control group (Figure **3**). According to the Poisson Generalized Estimating Equations model, the correlation between day 14 and month 6 data was 0.29 and the number of correct answers in the intervention group was not significantly different between the first and second assessments, suggesting good persistence of the effects of the educational session. In the control group, the number of correct answers improved significantly between the first and second assessments (*P*<10^-3^).

We found no significant between-group differences in replies to individual questions, even those explaining most of the between-group difference at the first assessment (“*Leftover antibiotics can be saved for later use*” and “If my child does not take all the antibiotic prescribed, some of the germs may survive”) (Table **7**). The proportions of correct answers to the question “Taking a few antibiotic doses is better than taking none at all” were still very low, about 50%, in both groups.

**Table 7:** Results of the knowledge questions at the second assessment after 6 months.

**Knowledge questions**	**Correct answer**	**Intervention group %(n=122)^a^**	**Control group %(n=118)^a^**	***P* value**
“If my child is feeling better, I sometimes save the rest of the antibiotic for the next time he get sick”	Disagree	92.6% (113)	94.9% (112)	0.46^c^
“Antibiotics are only effective if my child finishes all of them, even if my child’s symptoms are already gone”	Agree	96.7% (118)	92.4% (109)	0.14^b^
“I always follow the doctor’s instructions exactly when my child is taking an antibiotic?”	Agree	99.2% (121)	98.3% (116)	0.37^b^
“Left over antibiotics can be saved and used again”	Disagree	82.0% (100)	83.9% (99)	0.69^c^
“If my child doesn’t finish all of the antibiotic, some of the germs may survive”	Agree	91.8% (112)	88.1% (104)	0.34^c^
“Taking a few antibiotic doses is better than taking none at all”	Disagree	52.5% (64)	55.1% (65)	0.68^c^

^a^ percentages of correct answers, ^b^ exact Fisher test, ^c^ chi-square test

The number of phone calls per family for the second assessment ranged from 1 to 8. The mother completed the second assessment in 188 (78%) cases. The person interviewed for the second assessment was the same as for the first assessment in 89.1% of cases and had attended the education session in 89.4% of cases.

## Discussion

Our results demonstrate that a therapeutic education session on antibiotic use delivered in the PED improves parent satisfaction with information about antibiotic use and parent attitudes concerning judicious antibiotic use, compared to a control session on fever control. The effect of the session on antibiotic use might increase the likelihood of children receiving antibiotic therapy at home as prescribed by the physician.

The improved satisfaction about information on antibiotics received in the PED is encouraging, since dissatisfaction with explanations of medical problems or with treatment instructions given by the healthcare staff has a major influence on prescription filling by parents of children seen in PEDs [20,23,36]. In addition, parent satisfaction with care is considered a good surrogate for several important aspects of quality of care, such as appropriateness of the therapeutic regimen [21,24,37]. Both groups in our study received 30-minute face-to-face sessions, and satisfaction with information about fever control was better in the control group than in the intervention group, indicating that the operative factor was the content of the session and not the increased attention given to the family.

Failure to fill prescriptions given in the PED remains a major challenge [38]. According to the International Forum on Antibiotic Resistance colloquium held in 2002, educational interventions about antibiotics are more likely to be effective if they aim to change behaviors rather than only to provide information [16]. A household survey done in Great Britain in 2003 showed that better knowledge of antibiotics was independently associated with finishing a course of antibiotic as prescribed [39]. Our therapeutic education session on antibiotics was followed by improved attitudes about antibiotic use on day 14, compared to the control session, and the question “If my child does not take all the antibiotic prescribed, some of the germs may survive” was one of the two questions that showed the greatest improvements; the other was: “*Leftover antibiotics can be saved for later use*”. In a global survey of treatment non-adherence, awareness of the need to complete antibiotic courses and to discard any leftover antibiotics was associated with better adherence [32], a key factor for minimizing the emergence of bacterial resistance.

Antibiotic treatments for acute infectious diseases are brief. Decreasing their misuse requires an improvement in behavior at the time of prescription, during the acute illness of the child, which is likely to induce anxiety in the parents. The interview performed 6 months later gave us an indication of parent attitudes at a distance from the acute episode, when they may have recovered from any initial anxiety. Therefore, the second interview consisted only in the 6 questions on attitudes of antibiotic use.

The improved attitudes about antibiotic use in the control group at the second assessment after 6 months compared to the first assessment after 14 days may suggest that the anxiety associated with the acute illness in the child adversely affected performance on the attitudes questionnaire. This possibility would support the provision of education at the time of the acute illness. A study involving a third assessment at the time of a subsequent ARTI or urinary tract infection would further support this hypothesis if attitudes was again found to differ between the two groups.

Another hypothesis is that during the 6-month interval the families may have received further information on the proper use of antibiotics. However, no educational intervention targeting the public was conducted in France during the period extending from inclusion of the first patient to study completion (February 2009 to September 2011). However, we did not investigate the effect of the first assessment, which may have increased parent awareness about proper antibiotic use.

Interestingly, the overall proportion of correct answers to questions on attitudes about antibiotic use was high in both groups compared to that in the adherent group studied by Pechere et al. [32] (e.g., 77.5% in our intervention group, 66.2% in our control group, and 52.9% in the adherent group of the earlier study for the question “*Leftover antibiotics can be saved for later use*”). One possible explanation may be the nationwide public education campaign on antibiotic use conducted in France shortly before our study [40].

Although the study intervention was associated with improved attitudes about antibiotic use, there was no difference in the outcome of the infections after 14 days. The most common diagnosis in the study patients was acute otitis media, as expected since this disease is the leading reason for antibiotic prescription in PEDs [41]. However, for this disease, as well as for other upper respiratory tract infections, the systematic use of antibiotics is debatable, as clinical improvements seem related to antibiotic therapy in only a limited number of cases [42]. Therefore, we did not expect that our intervention could affect the clinical course. However, poor adherence to antibiotic treatment can increase the risk of carriage of non-penicillin-susceptible *Streptococcus pneumoniae* [32]. We nevertheless collected information on outcomes, because poorer outcomes might bias parent satisfaction with the antibiotic treatment.

At the time of the study, French guidelines recommended amoxicillin+clavulanate for the treatment of acute otitis media [41]. In a randomized controlled study, parents of children seen at a PED for acute otitis media were told to wait 2 or 3 days without giving antibiotic treatment; one group was given a prescription of antibiotic therapy to be filled if the symptoms persisted and the other was instructed to seek follow-up care if needed [43]. High levels of satisfaction were noted in both groups. However, this study did not involve education about antibiotic use [43]. In another randomized controlled trial, parents in a PED received education about antibiotics via either an animated video or the American Academy of Pediatrics pamphlet [44]. In neither group did the educational method involve interaction with healthcare professionals. Attitudes scores improved significantly in the education groups compared to the control group given no education, and the improvement lasted longer in the video group than in the pamphlet group [44]. Eight other educational interventions performed outside the PED have shown promising results that did not seem to occur at the expense of parent satisfaction [45]. One of them involved interaction between the parents and an educator [46]. Educational interventions to improve medication adherence for ARTIs can be categorized as educational, behavioral, organizational, or a mix of these components [31]. Some studies obtained improvements with interventions consisting of a single component. However, interventions that combine several components, as used in our study, seem more effective [31].

Importantly, the intervention conducted by a pharmacist was well accepted by the parents, although it extended their stay in the PED. Previous studies have demonstrated that pharmacist interventions in the emergency department can decrease treatment duration, healthcare costs, and medication errors, while at the same time improving antimicrobial stewardship [47,48].

Limitations of our study include the single-center design. The total numbers of patients with ARTIs and urinary tract infections seen in the PED during the study period were not available. A pharmacist was not available every day of the week or around the clock. We did not assess patient adherence to the prescribed treatment, and neither did we collect parameters known to affect adherence such as treatment regimen and antibiotic palatability [6]. Importantly, education was provided by a clinical pharmacist during a 30-minute face-to-face session. This facts limits the general applicability of the intervention, as the financial and human resources needed are unlikely to be available everywhere. However, the intervention could be either split into segments and delivered by nurses specialized in therapeutic education or conducted at the pharmacy during antibiotic dispensation.

That we were unable to obtain day-14 data for 21 (14%) intervention-group and 18 (12%) control-group patients may have affected our results. However, the intent-to-treat analysis of the primary outcome, in which missing data were replaced by unfavorable results, still showed better results in the intervention group. Although the trial was randomized, important predictors of attitude about antibiotic use were not measured such as parent educational level, previous exposure to antibiotic use, parent age, and socioeconomic status [32]. The efficacy of the randomization process in balancing these potential confounders is therefore uncertain, particularly among patients lost to follow-up. We cannot rule out a social desirability bias. However, the randomization process ensured that any differences between the groups were not systematic and then any difference between treatment groups was related to the intervention.

We included only children discharged with an antibiotic prescription. The objective of our intervention was not to decrease antibiotic use but, instead, to prevent the misuse of antibiotics by families, which can have a profound negative impact not only on the community, but also on individuals [6]. Finally, we did not include parents who did not speak French. Beliefs and attitudes toward antibiotics may vary across cultures.

## Conclusions

In summary, educational interventions delivered by clinical pharmacists in the PED were well accepted by families. An education session on antibiotic use was associated with high parent satisfaction and improved attitudes about judicious antibiotics use on day 14. However, no difference was found between the groups after 6 months. Our data suggest that educational interventions in PEDs may hold promise for decreasing the misuse of antibiotics.

## Supporting Information

Protocol S1 **Trial Protocol.**
(PDF)

Checklist S1 **CONSORT Checklist.**
(DOC)

## References

[1] Spellberg B, Guidos R, Gilbert D, Bradley J, Boucher HW et al. (2008) The epidemic of antibiotic-resistant infections: a call to action for the medical community from the Infectious Diseases Society of America. Clin Infect Dis 46: 155-164. doi:10.1086/524891. PubMed: 18171244. 18171244 10.1086/524891

[2] van de Sande-Bruinsma N, Grundmann H, Verloo D, Tiemersma E, Monen J et al. (2008) Antimicrobial drug use and resistance in Europe. Emerg Infect Dis 14: 1722-1730. doi:10.3201/eid1411.070467. PubMed: 18976555. 18976555 10.3201/eid1411.070467 PMC2630720

[3] Dowell SF, Schwartz B (1997) Resistant pneumococci: protecting patients through judicious use of antibiotics. Am Fam Physician 55: 1647-1658. PubMed: 9105195. 9105195

[4] Lieberman JM (2003) Appropriate antibiotic use and why it is important: the challenges of bacterial resistance. Pediatr Infect Dis J 22: 1143-1151. doi:10.1097/01.inf.0000101851.57263.63. PubMed: 14688589. 14688589 10.1097/01.inf.0000101851.57263.63

[5] Dajani AS (1996) Adherence to physicians’ instructions as a factor in managing streptococcal pharyngitis. Pediatrics 97: 976-980. PubMed: 8637785. 8637785

[6] Kardas P, Devine S, Golembesky A, Roberts C (2005) A systematic review and meta-analysis of misuse of antibiotic therapies in the community. Int J Antimicrob Agents 26: 106-113. doi:10.1016/S0924-8579(05)80302-4. PubMed: 16009535. 16009535 10.1016/j.ijantimicag.2005.04.017

[7] Grijalva CG, Nuorti JP, Griffin MR (2009) Antibiotic prescription rates for acute respiratory tract infections in US ambulatory settings. JAMA 302: 758-766. doi:10.1001/jama.2009.1163. PubMed: 19690308. 19690308 10.1001/jama.2009.1163 PMC4818952

[8] Gonzales R, Malone DC, Maselli JH, Sande MA (2001) Excessive antibiotic use for acute respiratory infections in the United States. Clin Infect Dis 33: 757-762. doi:10.1086/322627. PubMed: 11512079. 11512079 10.1086/322627

[9] Dommergues MA, Hentgen V (2012) Decreased paediatric antibiotic consumption in France between 2000 and 2010. Scand J Infect Dis 44: 495-501. doi:10.3109/00365548.2012.669840. PubMed: 22497317. 22497317 10.3109/00365548.2012.669840

[10] Finkelstein JA, Davis RL, Dowell SF, Metlay JP, Soumerai SB et al. (2001) Reducing antibiotic use in children: a randomized trial in 12 practices. Pediatrics 108: 1-7. doi:10.1542/peds.108.1.1. PubMed: 11433046. 11433046 10.1542/peds.108.1.1

[11] Dagan R, Lepage P (2009) Introduction: childhood respiratory diseases: management in an era of antibiotic resistance. Pediatr Infect Dis J 28: S119-S120. doi:10.1097/INF.0b013e3181b6d7d5. PubMed: 19918133. 19918133 10.1097/INF.0b013e3181b6d7d5

[12] Otters HB, van der Wouden JC, Schellevis FG, van Suijlekom-Smit LW, Koes BW (2004) Trends in prescribing antibiotics for children in Dutch general practice. J Antimicrob Chemother 53: 361-366. doi:10.1093/jac/dkh062. PubMed: 14729760. 14729760 10.1093/jac/dkh062

[13] Schindler C, Krappweis J, Morgenstern I, Kirch W (2003) Prescriptions of systemic antibiotics for children in Germany aged between 0 and 6 years. Pharmacoepidemiol Drug Saf 12: 113-120. doi:10.1002/pds.786. PubMed: 12642974. 12642974 10.1002/pds.786

[14] Price EL, Mackenzie TD, Metlay JP, Camargo CA Jr., Gonzales R (2011) A computerized education module improves patient knowledge and attitudes about appropriate antibiotic use for acute respiratory tract infections. Patient Educ Couns 85: 493-498. doi:10.1016/j.pec.2011.02.005. PubMed: 21392929. 21392929 10.1016/j.pec.2011.02.005

[15] World Health Organization (2001) WHO Global strategy for the containment of antimicrobial resistance. Available: http://www.who.int/csr/resources/publications/drugresist/WHO_CDS_CSR_DRS_2001_2_EN/en/. Accessed 1 March 2013.

[16] Finch RG, Metlay JP, Davey PG, Baker LJ, International Forum on Antibiotic Resistance Colloquium (2004); Educational interventions to improve antibiotic use in the community: report from the International Forum on Antibiotic Resistance (IFAR) colloquium (2002). Lancet Infect Dis 4: 44-53. 10.1016/s1473-3099(03)00860-0 14720568

[17] Goossens H, Guillemot D, Ferech M, Schlemmer B, Costers M et al. (2006) National campaigns to improve antibiotic use. Eur J Clin Pharmacol 62: 373-379. doi:10.1007/s00228-005-0094-7. PubMed: 16568344. 16568344 10.1007/s00228-005-0094-7

[18] Mangione-Smith R, McGlynn EA, Elliott MN, McDonald L, Franz CE et al. (2001) Parent expectations for antibiotics, physician-parent communication, and satisfaction. Arch Pediatr Adolesc Med 155: 800-806. doi:10.1001/archpedi.155.7.800. PubMed: 11434847. 11434847 10.1001/archpedi.155.7.800

[19] Wroth TH, Pathman DE (2006) Primary medication adherence in a rural population: the role of the patient-physician relationship and satisfaction with care. J Am Board Fam Med 19: 478-486. doi:10.3122/jabfm.19.5.478. PubMed: 16951297. 16951297 10.3122/jabfm.19.5.478

[20] Matsui D, Joubert GI, Dykxhoorn S, Rieder MJ (2000) Compliance with prescription filling in the pediatric emergency department. Arch Pediatr Adolesc Med 154: 195-198. doi:10.1001/archpedi.154.2.195. PubMed: 10665609. 10665609 10.1001/archpedi.154.2.195

[21] Ammentorp J, Mainz J, Sabroe S (2005) Parents’ priorities and satisfaction with acute pediatric care. Arch Pediatr Adolesc Med 159: 127-131. doi:10.1001/archpedi.159.2.127. PubMed: 15699305. 15699305 10.1001/archpedi.159.2.127

[22] Salazar ML, English TM, Eiland LS (2012) Caregivers’ baseline understanding and expectations of antibiotic use for their children. Clin Pediatr (Phila) 51: 632-637. doi:10.1177/0009922812439243. PubMed: 22399568. 22399568 10.1177/0009922812439243

[23] Boudreaux ED, O’Hea EL (2004) Patient satisfaction in the Emergency Department: a review of the literature and implications for practice. J Emerg Med 26: 13-26. doi:10.1016/j.jemermed.2003.04.003. PubMed: 14751474. 14751474 10.1016/j.jemermed.2003.04.003

[24] Laos CM, DiStefano MC, Cruz AT, Caviness AC, Hsu DC et al. (2012) Mobile pediatric emergency response team: patient satisfaction during the novel H1N1 influenza outbreak. Acad Emerg Med 19: 274-279. doi:10.1111/j.1553-2712.2012.01289.x. PubMed: 22435859. 22435859 10.1111/j.1553-2712.2012.01289.x

[25] Spahr CD, Flugstad NA, Brousseau DC (2006) The impact of a brief expectation survey on parental satisfaction in the pediatric emergency department. Acad Emerg Med 13: 1280-1287. doi:10.1111/j.1553-2712.2006.tb00291.x. PubMed: 17099193. 17099193 10.1197/j.aem.2006.06.059

[26] Bordley WC (2002) Outcomes research and emergency medical services for children: domains, challenges, and opportunities. Ambul Pediatr 2: 306-310. doi:10.1367/1539-4409(2002)002. PubMed: 12135405. 12135405 10.1367/1539-4409(2002)002<0306:oraems>2.0.co;2

[27] Rega PP, Roberts SM, Khuder S, Boardley D, Brickman K et al. (2012) The delivery of a health promotion intervention by a public health promotion specialist improves patient satisfaction in the emergency department. Acad Emerg Med 19: 313-317. doi:10.1111/j.1553-2712.2012.01293.x. PubMed: 22435864. 22435864 10.1111/j.1553-2712.2012.01293.x

[28] World Health Organization (1998) Therapeutic patient education. Available: http://www.euro.who.int/__data/assets/pdf_file/0007/145294/E63674.pdf. Accessed 1 March 2013.

[29] Autorité de Santé Haute (2007) Therapeutic patient education (TPE) Definition, goals, and organisation. Available: http://www.has-sante.fr/portail/upload/docs/application/pdf/2008-12/therapeutic_patient_education_tpe_-_offering_and_providing_tpe_-_quick_reference_guide.pdf. Accessed 8 February 2013.

[30] Barrows HS, Tamblyn RM (1980) Problem-Based Learning: An Approach to Medical Education. New York, NY: Springer Verlag Publishing Company. 206pp.

[31] Wu YP, Roberts MC (2008) A meta-analysis of interventions to increase adherence to medication regimens for pediatric otitis media and streptococcal pharyngitis. J Pediatr Psychol 33: 789-796. doi:10.1093/jpepsy/jsn009. PubMed: 18296457. 18296457 10.1093/jpepsy/jsn009

[32] Pechère JC, Hughes D, Kardas P, Cornaglia G (2007) Non-compliance with antibiotic therapy for acute community infections: a global survey. Int J Antimicrob Agents 29: 245-253. doi:10.1016/S0924-8579(07)70779-3. PubMed: 17229552. 17229552 10.1016/j.ijantimicag.2006.09.026

[33] Isaacman DJ, Khine H, Losek JD (1997) A simple intervention for improving telephone contact of patients discharged from the emergency department. Pediatr Emerg Care 13: 256-258. doi:10.1097/00006565-199708000-00004. PubMed: 9291512. 9291512 10.1097/00006565-199708000-00004

[34] Sixsmith DM, Weissman L, Constant F (1997) Telephone follow-up for case finding of domestic violence in an emergency department. Acad Emerg Med 4: 301-304. doi:10.1111/j.1553-2712.1997.tb03553.x. PubMed: 9107330. 9107330 10.1111/j.1553-2712.1997.tb03553.x

[35] Altman DG (2009) Missing outcomes in randomized trials: addressing the dilemma. Open Med 3: e51-e53. PubMed: 19946393. 19946393 PMC2765768

[36] Thomas EJ, Burstin HR, O’Neil AC, Orav EJ, Brennan TA (1996) Patient noncompliance with medical advice after the emergency department visit. Ann Emerg Med 27: 49-55. doi:10.1016/S0196-0644(96)70296-2. PubMed: 8572448. 8572448 10.1016/s0196-0644(96)70296-2

[37] Hall JA, Roter DL, Katz NR (1988) Meta-analysis of correlates of provider behavior in medical encounters. Med Care 26: 657-675. doi:10.1097/00005650-198807000-00002. PubMed: 3292851. 3292851 10.1097/00005650-198807000-00002

[38] Rosman SL, Dorfman D, Suglia SF, Humphrey C, Silverstein M (2012) Predictors of prescription filling after visits to the pediatric emergency department. Pediatr Emerg Care 28: 22-25. doi:10.1097/PEC.0b013e31823ed6e4. PubMed: 22193695. 22193695 10.1097/PEC.0b013e31823ed6e4

[39] McNulty CA, Boyle P, Nichols T, Clappison P, Davey P (2007) Don’t wear me out--the public’s knowledge of and attitudes to antibiotic use. J Antimicrob Chemother 59: 727-738. doi:10.1093/jac/dkl558. PubMed: 17307770. 17307770 10.1093/jac/dkl558

[40] Sabuncu E, David J, Bernède-Bauduin C, Pépin S, Leroy M et al. (2009) Significant reduction of antibiotic use in the community after a nationwide campaign in France, 2002-2007. PLOS Med 6: e1000084 PubMed: 19492093. 19492093 10.1371/journal.pmed.1000084 PMC2683932

[41] Angoulvant F, Skurnik D, Bellanger H, Abdoul H, Bellettre X et al. (2012) Impact of implementing French antibiotic guidelines for acute respiratory-tract infections in a paediatric emergency department, 2005-2009. Eur J Clin Microbiol Infect Dis 31: 1295-1303. doi:10.1007/s10096-011-1442-4. PubMed: 22002230. 22002230 10.1007/s10096-011-1442-4

[42] Azria R, Barry B, Bingen E, Cavallo JD, Chidiac C et al. (2012) Antibiotic stewardship. Med Mal Infect 42: 460-487. doi:10.1016/j.medmal.2012.02.004.

[43] Chao JH, Kunkov S, Reyes LB, Lichten S, Crain EF (2008) Comparison of two approaches to observation therapy for acute otitis media in the emergency department. Pediatrics 121: e1352-1356 PubMed: 18450878. 18450878 10.1542/peds.2007-2278

[44] Schnellinger M, Finkelstein M, Thygeson MV, Vander Velden H, Karpas A et al. (2010) Animated video vs pamphlet: comparing the success of educating parents about proper antibiotic use. Pediatrics 125: 990-996. doi:10.1542/peds.2009-2916. PubMed: 20385634. 20385634 10.1542/peds.2009-2916

[45] Andrews T, Thompson M, Buckley DI, Heneghan C, Deyo R et al. (2012) Interventions to influence consulting and antibiotic use for acute respiratory tract infections in children: a systematic review and meta-analysis. PLOS ONE 7: e30334. doi:10.1371/journal.pone.0030334. PubMed: 22299036. 22299036 10.1371/journal.pone.0030334 PMC3267713

[46] Alder S, Trunnell E, White G, Lyon J, Reading J et al. (2005) Reducing Parental Demand for Antibiotics by Promoting Communication Skills. Am J Health Educ 36: 132-139. doi:10.1080/19325037.2005.10608174.

[47] May L, Cosgrove S, L’Archeveque M, Talan DA, Payne P et al. (2013) A Call to Action for Antimicrobial Stewardship in the Emergency Department: Approaches and Strategies. Ann Emerg Med 62: 69-77. doi:10.1016/j.annemergmed.2012.09.002. PubMed: 23122955. 23122955 10.1016/j.annemergmed.2012.09.002 PMC3872779

[48] Cesarz JL, Steffenhagen AL, Svenson J, Hamedani AG (2013) Emergency department discharge prescription interventions by emergency medicine pharmacists. Ann Emerg Med 61: 209-214. doi:10.1016/j.annemergmed.2012.04.011. PubMed: 22633338. 22633338 10.1016/j.annemergmed.2012.04.011 PMC4145851

