# Supplementary material for: Randomized Controlled Trial of Parent Therapeutic Education on Antibiotics to Improve Parent Satisfaction and Attitudes in a Pediatric Emergency Department
Source: PLoS One. 2013 Sep 26;8(9):e75590. doi: 10.1371/journal.pone.0075590 (PMC3784452; doi:10.1371/journal.pone.0075590)
Supplement: Protocol S1 — Trial Protocol. (PDF) [file pone.0075590.s001.pdf]

Protocole « Achéen » (Antibiotique chez l'Enfant)

**Education thérapeutique sur la prise d'antibiotique buvable chez des enfants de moins de 6 ans aux urgences pédiatriques de l'hôpital Robert Debré**

PROTOCOLE DE RECHERCHE EN SOINS COURANTS

Version N°1 du 05/09/2008
N° ID RCB : 2008-A00892-53

Investigateur Coordonnateur

Dr François ANGOULVANT
Hôpital Robert Debré
48 Bd Sérurier
75935 PARIS cedex 19
Tél : 01 40 03 38 90 – 01 40 03 22 72
Fax : 01 40 03 47 44
e.mail : francois.angoulvant@rdb.aphp.fr

UEC Robert Debré

Adyla YACOUBI
48 Bd Sérurier
75935 Paris cedex 19
Tél : 01 40 03 23 47
Fax : 01 40 03 24 85
e.mail : adyla.yacoubi@rdb.aphp.fr

Méthodologiste

Pr Corinne ALBERTI
U.E.C., Hôpital Robert Debré
48 Bd Sérurier
75935 PARIS cedex 19
Tél : 01 40 03 23 45
Fax : 01 40 03 24 85
e-mail : corinne.alberti@rdb.aphp.fr

Page de SIGNATURE D'UN PROTOCOLE de recherche en soins courants

Titre : « Education thérapeutique sur la prise d'antibiotique buvable chez des enfants de moins de 6 ans aux urgences pédiatriques de l'hôpital Robert Debré – Paris »
Version N° 1 du : 22/09/08

La recherche sera conduite conformément au protocole et aux dispositions législatives et réglementaires en vigueur.

L'investigateur coordonnateur :
Dr François Angoulvant
Service
Hôpital Robert Debré
Paris

La recherche a reçu un avis favorable du CPP île de France N°2 en date du / / .

TABLE DES MATIÈRES

1. RÉSUMÉ SYNOPTIQUE 4

2. RATIONNEL DE LA RECHERCHE 6

3. JUSTIFICATION DE L'ETUDE : 7

4. OBJECTIFS 8

5. PLAN EXPERIMENTAL 8

5.1. PLAN DE L'ETUDE 8
5.1.1. Type d'étude 8
5.1.2. Durée de l'étude : L'étude devrait se dérouler sur deux ans. 9
5.1.3. Lieu 9
5.1.4. Nombre prévu de personnes et justification 9
5.1.5. Personnel impliqué 9
5.1.6. Matériel utilisé 9
5.2. POPULATION ETUDIEE 10
5.2.1. Critères d'inclusion 10
5.2.2. Critères de non inclusion 10
5.3. METHODOLOGIE DE LA RECHERCHE: 10
5.4. TIRAGE AU SORT: 10
5.5. CRITERES D'EVALUATION PRINCIPAUX ET SECONDAIRES 10
5.6. DEROULEMENT DE LA RECHERCHE 12
5.7. ARRET DEFINITIF OU TEMPORAIRE DE PARTICIPATION D'UN SUJET 13

6. MODALITES DE RECRUTEMENT ET D'INFORMATION DES PERSONNES 13

7. GESTION DES EVENEMENTS INDESIRABLES 13

8. GESTION DES DONNEES 13

8.1. CAHIERS D'OBSERVATION : 13
8.2. IDENTIFICATION DES DONNEES QUI SERONT CONSIDEREES COMME DONNEES-SOURCE ..... **ERREUR ! SIGNET NON DEFINI.**
8.3. TRAITEMENT DES DONNEES ET CONSERVATION DES DOCUMENTS ET DES DONNEES 13

9. ASPECTS STATISTIQUES 14

9.1. JUSTIFICATION DE LA TAILLE DE L'ECHANTILLON 14
9.2. DESCRIPTION DES METHODES STATISTIQUES PREVUES Y COMPRIS DU CALENDRIER DES ANALYSES INTERMEDIAIRES PREVUES 14

10. ASPECTS ETHIQUES ET LEGAUX 14

10.1. OBLIGATIONS LEGALES (ROLE DU GESTIONNAIRE, CPP, CCTIRS, CNIL) 14
10.2. MODIFICATIONS DE LA RECHERCHE 15

11. BIBLIOGRAPHIE 16

12. ANNEXES 17

1. RÉSUMÉ SYNOPTIQUE

**Titre**	Education thérapeutique sur la prise d'antibiotique buvable chez des enfants de moins de 6 ans aux urgences pédiatriques de l'hôpital Robert Debré – Paris
**Gestionnaire**	Assistance Publique – Hôpitaux de Paris
**Investigateur Coordonnateur**	Dr François Angoulvant
**Population concernée**	Enfant âgé de 1 mois à 6 ans
**Nature du soin évalué**	Education thérapeutique
**Nombre de centres**	Monocentrique
**Nombre de patients prévus**	300
**Objectif principal**	Evaluer la satisfaction après un programme d'éducation thérapeutique aux urgences pédiatriques sur la prise d'antibiotique buvable chez des enfants âgés de 1 mois à 6 ans.
**Objectifs secondaires**	Connaissance des parents sur le bon usage des antibiotiques
**Critères d'inclusion**	Enfants âgés de 1 mois à 6 ans, de famille francophone, chez qui sera prescrit un antibiotique oral buvable pour une durée de 5 à 10 jours inclus, dans le cadre d'une infection respiratoire ou urinaire aiguë.<br><br>Présence des parents lors de la consultation aux urgences, et joignables par téléphone par la suite.<br><br>Non-opposition des détendeurs de l'autorité parentale à la participation de l'enfant à l'étude.<br><br>Patient affilié à un régime de sécurité sociale ou CMU*<br>*CMU* : Couverture médicale universelle*
**Critères de non inclusion**	- Présence d'une pathologie chronique altérant la prise du médicament (ex : troubles de la déglutition, sonde gastrique…)<br>- Famille non francophone.<br>- Non joignable par téléphone ou refus d'être recontacté<br>- Allergie (suspectée ou avérée) à l'antibiotique prescrit
**Critères d'évaluation**	L'évaluation de l'impact de l'ETP sur la prise d'antibiotique buvable chez des enfants de moins de 6 ans se fera par une interview téléphonique à J14.<br><br>*<u>Le critère de jugement principal est la satisfaction des parents sur l'information reçue concernant le traitement antibiotique</u>*<br><br>En effet, la satisfaction des parents dans le champ de la pédiatrie peut être considérée comme un outil d'évaluation de la qualité de la prise en charge [1, 2] : la satisfaction des parents est corrélée à l'amélioration de la santé de l'enfant, via :<br><br>- La compréhension des informations médicales<br>- L'observance du traitement.<br>*<u>Les critères de jugements secondaires sont les suivants :</u>*<br><br>Les connaissances sur l'usage des antibiotiques. Lors de l'interview à J14. On les contactera à nouveau à 6 mois pour

	tester à nouveau les connaissances sur l'usage des antibiotiques. En effet les connaissances des patients sur le bon usage des antibiotiques sont corrélées à l'observance [3-5].
**Méthodologie statistique**	Statistique descriptives des caractéristiques socio-démographiques, médicales, environnementales. Utilisation d'échelle de likert dichotomisées pour les critères de jugement et comparaison par test du chi2. Analyse en intention de traiter.
**Durée de participation d'un patient**	6 mois
**Calendrier de l'étude**	- Une seule visite<br>- Appel téléphonique à J14 et à 6 mois

2. RATIONNEL DE LA RECHERCHE

L'observance thérapeutique est primordiale dans le domaine de l'antibiothérapie car elle est reliée à la guérison, au risque de rechute et d'infection récurrente, à l'augmentation de la résistance des bactéries [6]. Ainsi certains suggèrent que la mauvaise observance thérapeutique dans les pathologies infectieuses de l'enfant contribue au développement de la résistance aux antibiotiques [6]. L'observance thérapeutique est un processus dynamique qui fluctue au cours du temps, en fonction de différentes composantes (cliniques, thérapeutiques, psycho-sociologiques, matérielles, etc.), interagissant entre elles [6]. Les pathologies infectieuses du tractus respiratoire et ORL de l'enfant sont fréquentes et l'antibiothérapie de ces pathologies constitue un enjeu de santé publique. Ainsi en octobre 2005 l'Agence Française de Sécurité Sanitaire des Produits de Santé (AFSSAPS) a actualisé ses recommandations sur les modalités et les indications de l'antibiothérapie des voies respiratoires.

Plusieurs facteurs influent sur l'observance, certains dépendent de la nature et des caractéristiques du traitement [7-9]. D'autres dépendent du patient, de sa famille, du médecin et de la communication entre ces différents acteurs [1, 5, 10].

Les premières interventions visant à améliorer l'observance thérapeutique dans les infections des voies respiratoires sont anciennes [11]. Mais depuis 20 ans la recherche sur ce sujet semble s'être arrêtée alors que le problème de résistance aux antibiotiques ne fait que croître [6].

Dans le même temps un autre domaine prend de l'essor, celui de l'éducation thérapeutique.

Quelque soit l'âge du patient, l'éducation thérapeutique du patient doit « *permettre aux patients d'acquérir et de conserver les compétences les aidant à vivre de manière optimale avec leur maladie* » (Définition OMS, cité par D'Ivernois [12]), et donc de l'aider à devenir acteur de sa santé.

La prise en charge du patient-enfant présente cependant certaines particularités auxquelles l'éducation thérapeutique (ETP) doit s'adapter. L'enfant est en effet un être en développement, dépendant des adultes et de son entourage : un programme d'éducation thérapeutique du patient destiné à l'enfant ne peut donc pas être calqué sur les programmes destinés aux adultes [13].

Dans sa mise en œuvre (choix des outils et méthodes pédagogiques et/ou évaluatives…) le soignant éducateur doit donc non seulement tenir compte de l'âge de l'enfant, de sa maturité, mais aussi de son environnement familial et extra-familial.

L'ETP s'adresse ainsi au patient, sa famille et à tous les acteurs de soins qui gravitent autour de l'enfant (grands-parents, nourrice, enseignants….).

Deux extrêmes sont identifiés :

- le nourrisson, dont la dépendance aux adultes est totale : l'éducation s'adresse donc essentiellement aux parents,
- l'adolescent, dont les parents assurent un encadrement plus ou moins distant, fonction de son autonomie : l'éducation sera alors recentrée sur ce dernier.

Au total, l'ETP permet l'acquisition de compétences (partagées entre l'enfant et son entourage) permettant une autonomie familiale dans la gestion quotidienne de la maladie.

L'amélioration de l'observance dans le cadre de l'antibiothérapie est donc liée d'une part à l'amélioration des connaissances pratiques mais aussi à l'amélioration des connaissances sur le bon usage des antibiotiques.

La mise en place d'un programme d'éducation thérapeutique dans le service des urgences pédiatriques de l'hôpital Robert Debré s'inscrit dans le contexte d'une prise en charge aiguë et non dans la chronicité d'une pathologie. S'agissant d'éduquer l'enfant et ses parents sur la bonne prise des antibiotiques, cette éducation vise néanmoins à leur permettre d'acquérir, ou de conserver des compétences qu'ils pourront être amenés à mobiliser tout au long de leur vie. En effet on peut estimer que dans sa vie un patient sera à plusieurs reprises confronté à une prescription d'antibiotique pour lui-même ou pour ses proches.

3. JUSTIFICATION DE L'ETUDE :

En 2006, un Accord-cadre national relatif au bon usage des antibiotiques dans les établissements de santé a été mis en place entre les hôpitaux et la Caisse Nationale d'Assurance Maladie. Son objectif est d'améliorer la qualité de la prescription des antibiotiques à l'hôpital.

Les protocoles médicaux du service des urgences suivent les recommandations officielles et notamment les recommandations de l'AFSSAPS sur les infections respiratoires et urinaires. Et si le bon usage des antibiotiques repose notamment sur le respect des recommandations officielles, le suivi des bactéries multirésistantes, etc., il passe aussi par le conseil et l'éducation des patients vis-à-vis des antibiotiques.

Dans les recommandations sur « l'antibiothérapie par voie générale en pratique courante dans les infections respiratoires hautes de l'adulte et l'enfant » publiées en 2005, l'AFSSAPS insiste sur la nécessaire sensibilisation et éducation des patients pour faciliter l'observance [14].

Le service des urgences a décidé de mettre en place un programme d'éducation thérapeutique sur la prise d'antibiotique buvable chez les enfants âgés de 1 mois à 6 ans aux urgences pédiatriques.
Certaines études [3, 8, 15] ont démontré que l'observance aux traitements prescrits aux urgences, dont les antibiotiques, est relativement faible : 50 à 80% . Il est intéressant de noter que ces chiffres rejoignent les valeurs observées lorsqu'on évalue l'observance des traitements prescrits dans le cadre des maladies chroniques… Par ailleurs, d'autres travaux [16, 17] ont démontré que la famille ne se procurait pas le traitement antibiotique prescrit aux urgences dans 7 à 35% des cas, malgré une forte demande d'une prescription antibiotique [18, 19].

Dans le cadre des traitements antibiotiques, les facteurs influençant les comportements d'observance sont liés :

- au patient et à sa famille :
  - une faible connaissance sur le traitement antibiotique [5, 10], notamment liée à un manque d'information (information incomplète, inadéquate) sur les modalités de prise du traitement [7].
  - l'âge [8, 9], les difficultés des familles à administrer le traitement aux enfants ou aux nourrissons [7].
- Au traitement prescrit [7, 9]:
  - la durée du traitement, le nombre de prises, les effets indésirables tels que les vomissements.
  - le coût du traitement.
  - le goût.
  - les erreurs dans la reconstitution du médicament ou dans son mode de conservation.

Une meilleur connaissance du bon usage des antibiotiques par les patients et leurs familles devrait améliorer l'observance au traitement antibiotique et en limiter le mésusage [5, 20, 21]. La connaissance du bon usage des antibiotiques est associée à une meilleur observance ainsi que l'ont notamment démontré Pechère et Coll. [5] qui ont interviewé plus de 4000 patients lors d'une étude multicentrique internationale.

On peut schématiser le principe de l'étude selon la figure suivante

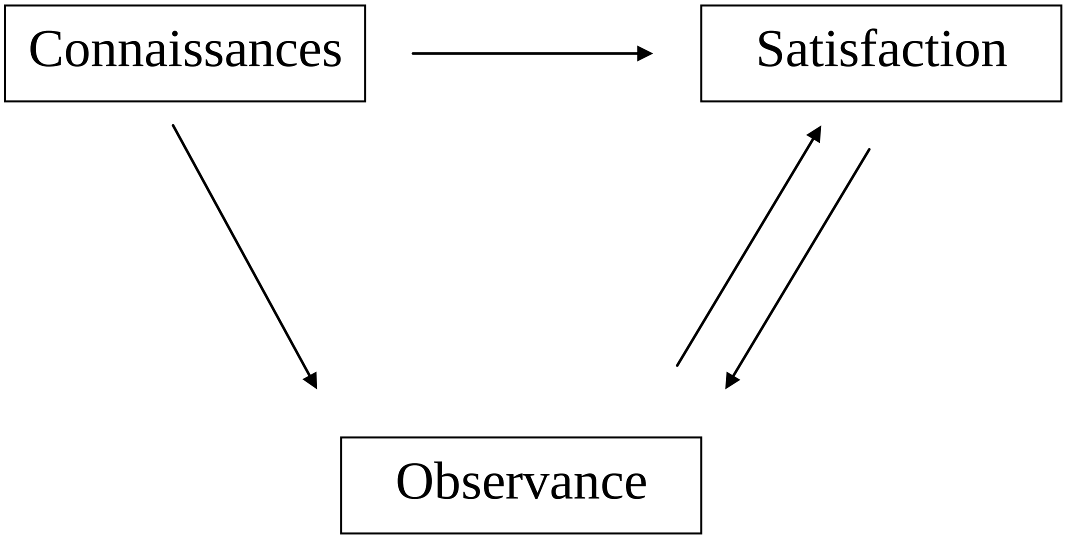

Afin de démontrer le bénéfice de l'éducation thérapeutique sur la prise d'antibiotique buvable chez des enfants âgés de 1 mois à 6 ans aux urgences pédiatriques, la mise en place de ce programme se fera dans le cadre d'un projet de recherche clinique.

Ce projet a reçu le soutien scientifique du Groupe de Pathologie Infectieuse Pédiatrique (GPIP). De plus il s'inscrit dans l'actualité puisqu'un des thèmes de la Présidence de la France du Conseil de l'Union Européenne à partir de juillet 2008 sera l'antibiothérapie.

4. OBJECTIFS

Evaluer un programme d'éducation thérapeutique aux urgences pédiatriques sur la prise d'antibiotique buvable chez des enfants âgés de 1 mois à 6 ans en mesurant la satisfaction et les connaissance des familles pour le traitement prescrit.

5. PLAN EXPERIMENTAL

5.1. Plan de l'étude

5.1.1. Type d'étude

Il s'agit d'une étude prospective randomisée contrôlée monocentrique avec groupe parallèle, en aveugle du patient et de l'évaluateur.

Le bras évalué recevra une éducation thérapeutique sur la prise d'antibiotique buvable chez des enfants âgés de 1 mois à 6 ans. Le bras contrôle recevra une éducation thérapeutique sur la prise en charge de la fièvre chez des enfants âgés de 1 mois à 6 ans. L'évaluation se fera par deux interviews téléphoniques, à 14 jours et 6 mois plus tard.

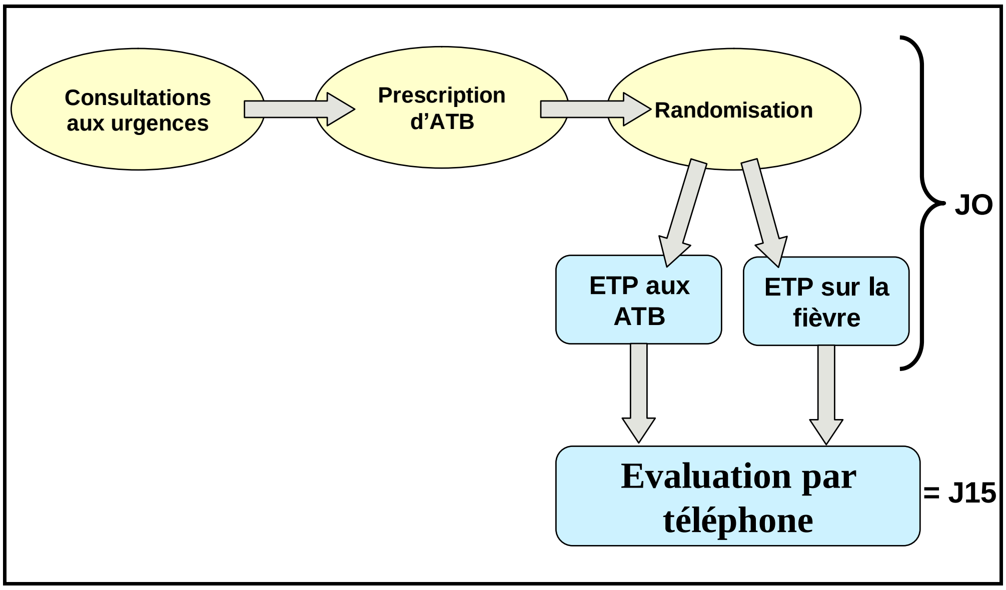

Figure 1 : Méthodologie suivie pour l'évaluation

5.1.2. Durée de l'étude : L'étude devrait se dérouler sur deux ans.

Inclusion pendant 1 an du 1er décembre 2008 au 30 novembre 2009.

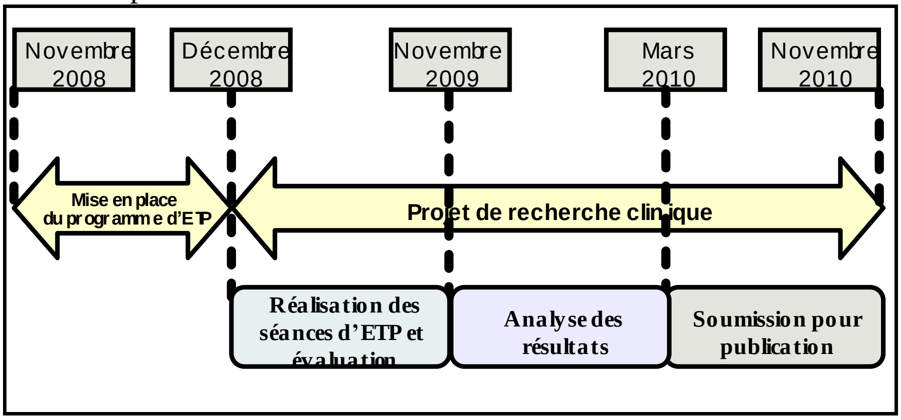

Figure 2: calendrier du programme d'éducation thérapeutique (Urgences pédiatriques)

5.1.3. Lieu

La mise en place de ce programme d'éducation thérapeutique aura lieu dans le service des urgences pédiatriques du CHU Robert Debré, 66 000 entrées par an sont dénombrées.

5.1.4. Nombre prévu de personnes et justification

Il est prévu d'inclure 300 enfants soit 150 par bras. Cf chapitre 9.1.

5.1.5. Personnel impliqué

Service de Pharmacie : Dr Françoise Brion (PUPH), Dr Olivier Bourdon (MCUPH), Anne Rouault (interne en pharmacie, en année médaille d'or), Laurence Spiesser (interne en pharmacie)

Service d'Accueil des Urgences : Dr François Angoulvant (PH), Pr Jean-Christophe Mercier (PUPH)

Service d'Epidémiologie Clinique : Pr Corinne Alberti (PUPH), Adyla YACOUBI (ARC)

5.1.6. Matériel utilisé

L'ordonnance sera pré-formatée (nom de l'antibiotique et de l'antipyrétique, galénique, dosage, nombre de prise, durée du traitement) et réalisée à l'aide du logiciel URQUAL®. Ce logiciel gère informatiquement les dossiers des patients : nom, prénom, coordonnées de l'enfant et sa famille, dossier médical (anamnèse, examen clinique, diagnostic, traitement)

5.2. Population étudiée

5.2.1. Critères d'inclusion

Il s'agit d'enfants âgés de 1 mois à 6 ans, de famille francophone, chez qui sera prescrit un antibiotique oral buvable pour une durée de 5 à 10 jours inclus, dans le cadre d'une infection respiratoire ou urinaire aiguë.

Les parents devront être présents lors de la consultation aux urgences, et joignables par téléphone. L'accord des détendeurs de l'autorité parentale sera préalable à l'inclusion de l'enfant.

Tout patient doit être affilié à un régime de sécurité sociale ou CMU.

5.2.2. Critères de non inclusion

- Présence d'une pathologie chronique altérant la prise du médicament (ex : troubles de la déglutition, sonde gastrique…)
- Famille non francophone
- Famille non joignable par téléphone ou refus d'être recontactée
- Refus de participation (Au moins l'un des deux parents ou le tuteur légal doit être présent et doit avoir été informé de l'étude)
- Allergie (suspectée ou avérée) à l'antibiotique prescrit

5.3. Méthodologie de la recherche:

Il s'agit d'une étude randomisée contrôlée avec deux groupes parallèles.
Tous les enfants consultant aux urgences de l'hôpital Robert Debré sont potentiellement incluables dans l'étude. Les inclusions se dérouleront pendant la journée (9h00 – 18h00) et pendant les jours de semaines (lundi au vendredi inclus). Si le rythme des inclusions s'avère trop lent, la période des inclusions pourrait être étendue aux soirs et aux jours de week-end.
Dès qu'il identifiera un patient potentiellement incluable, le médecin des urgences préviendra un des médecins investigateurs, à la fin de la consultation médicale. Il remettra ensuite les ordonnances à la famille.
Le médecin investigateur vérifiera le respect des critères d'inclusion et de non inclusion. Il procédera à l'inclusion avec l'accord de la famille. Immédiatement après, l'éducateur réalisera la randomisation en se connectant par intranet puis la séance d'éducation selon le bras déterminé.
La séance d'éducation débutera par le recueil des données (cf Annexe 2).
L'interview téléphonique se déroulera entre J14 et J17. Il y aura au moins 3 essais sur 2 jours à partir des numéros de téléphone communiqués à J1.
Idem à 6 mois.

5.4. Tirage au sort:

La randomisation sera centralisée à l'Unité d'Epidémiologie Clinique (UEC) et sera effectuée par blocs de tailles variables. Le jour de l'inclusion le médecin investigateur se connecte sur Intranet afin d'effectuer la randomisation en ligne à l'aide d'une application développée par Hakim Laouamri bio-informaticien. L'investigateur reçoit par e-mail le résultat de la randomisation et un double est envoyé au Dr Corinne Alberti, UEC, Robert Debré.

5.5. Critères d'évaluation principaux et secondaires

L'évaluation du programme devra aborder notamment la mise en place, le déroulement d'une séance d'ETP, l'intégration de ce programme au sein du service, l'éducateur, le patient et sa famille.

L'évaluation de l'impact de l'ETP sur la prise d'antibiotique buvable chez des enfants âgés de 1 mois à 6 ans se fera par une interview téléphonique entre J14 et J17 et à 6 mois plus tard. (cf tableau 1)

Le critère de jugement principal sera la satisfaction des parents sur les informations reçues sur le traitement antibiotique

En effet, la satisfaction des parents dans le champ de la pédiatrie peut être considérée comme un outil d'évaluation de la qualité de la prise en charge [1, 2] : la satisfaction des parents est corrélée à l'amélioration de la santé de l'enfant, via :

- La compréhension des informations médicales
- L'observance du traitement.

Les critères de jugements secondaires :

- La satisfaction des parents sur les informations reçues pour traiter la fièvre.
- Concernent les connaissances des parents sur le bon usage des antibiotiques :
- Evolution de l'état de santé de l'enfant

Les paramètres recueillis lors des l'interviews téléphoniques sont récapitulés dans le tableau 1 :

1. Est-ce que votre enfant est guéri ? ❒ oui ❒ non,<br>Si non est-ce qu'un autre antibiotique lui a été prescrit ? ❒ oui ❒ non
2. Quel est votre degré de satisfaction sur les informations données à l'hôpital sur l'antibiotique ? Etes-vous : (Echelle de satisfaction) ? |__|
3. Quel est votre degré de satisfaction sur les informations données à l'hôpital pour vous aider à traiter la fièvre ? |__| (Echelle de satisfaction)
4. Quel a été votre degré de difficultés pour faire boire l'antibiotique au bon moment à l'enfant ? |__| (Echelle de difficulté)
5. Quel est votre degré d'accord avec la phrase suivante : «Lorsque mon enfant est guéri, je garde le reste des antibiotiques pour la prochaine fois qu'il sera malade » |__| (Echelle d'accord)
6. Quel est votre degré d'accord avec la phrase suivante : «Même si les symptômes ont disparu mon enfant doit quand même finir l'antibiotique car l'antibiotique est efficace uniquement si on prend toutes les doses ? » Etes vous … (Echelle d'accord) |__|
7. Quel est votre degré d'accord avec la phrase suivante : «Lorsque mon enfant prend un antibiotique je suis toujours exactement les instructions du médecin ? » Etes vous … (Echelle d'accord) |__|
8. Quel est votre degré d'accord avec la phrase suivante : «Je garde les antibiotiques qui n'ont pas été pris pour une autre fois ? » Etes vous … (Echelle d'accord) |__|
9. Quel est votre degré d'accord avec la phrase suivante : «Si mon enfant ne prend pas toutes les doses d'antibiotiques des microbes peuvent survivre ? » Etes vous … (Echelle d'accord) |__|
10. Quel est votre degré d'accord avec la phrase suivante : « Prendre quelques doses d'antibiotique est mieux que de ne pas en prendre du tout ? » Etes vous … (Echelle d'accord) |__|

Tableau 1 : Questionnaire téléphonique.

Tous les critères à l'exception de la question 1 sont basés sur des échelles de Likert à 5 points :

Echelle de Satisfaction	Echelle de Difficulté	Echelle d'Accord
5 : Très satisfait	5 : Pas du tout	5 : Complètement d'accord
4 : Plutôt satisfait	4 : Modérée	4 : Plutôt en accord
3 : Ni satisfait, ni insatisfait	3 : Assez difficile	3 : Ni en accord, ni en désaccord
2 : Plutôt insatisfait	2 : Très difficile	2 : Plutôt en désaccord
1 : Très insatisfait	1 : Presque impossible	1 : Pas du tout d'accord

Les questions sur la connaissance du bon usage des antibiotiques ont déjà été validées lors d'enquêtes multicentriques internationales [5].

5.6. Déroulement de la recherche

Les séances d'ETP se dérouleront immédiatement après la fin de la consultation par le médecin prescripteur, une fois l'accord de la famille obtenu. Elles auront lieu dans le même box de consultation. Les ordonnances remises au patient et/ou aux parents serviront de support à la séance.

les méthodes et outils pédagogiques comporteront un support papier et des exemples pratiques à l'aide de flacon de démonstration. Il est prévu que les parents goûtent l'antibiotique prescrit, pour leur démontrer l'importance du goût de l'enfant dans la bonne prise du traitement.

Objectif de la séance d'éducation thérapeutique sur la prise d'un antibiotique buvable pour l'enfant (selon l'âge) et les adultes l'accompagnant :

- Comprendre le rôle du traitement ATB par rapport à la maladie diagnostiquée
- Comprendre les modalités du traitement
- Maîtriser les techniques de reconstitution du médicament
- Maîtriser l'administration du médicament chez l'enfant (éviter les crachats, masquer le goût…)
- Connaître les effets indésirables pouvant survenir
- Gérer la survenue des effets indésirables liés au traitement
- Expliquer à l'entourage de l'enfant l'importance du traitement, son rôle et les modalités de prise.
- Définir ce qu'est un générique (la prescription d'ATB sera rédigée en Dénomination Commune Internationale)
- Etre capable de préciser à la pharmacie d'officine le goût que le générique dispensé doit avoir.

Objectif de la séance d'éducation thérapeutique sur la prise en charge de la fièvre pour l'enfant (selon l'âge) et les adultes l'accompagnant :

- Comprendre le mécanisme de la fièvre
- Comprendre les modalités du traitement antipyrétique (médicamenteux et non médicamenteux) et la réalisation des mesures physiques (utilisation du thermomètre, valeurs cibles, interprétation…)
- Savoir mettre en pratique ces modalités
- Maîtriser l'administration du médicament chez l'enfant
- Expliquer à l'entourage de l'enfant l'importance de la prise en charge, les modalités de prise du traitement et de réalisation des mesures physiques.

5.7. Arrêt définitif ou temporaire de participation d'un sujet

Il n'est pas prévu de sortie d'étude, en dehors de l'opposition du patient ou de sa famille et des perdus de vue.

En cas d'opposition les données ne seront pas analysées.

6. MODALITES DE RECRUTEMENT ET D'INFORMATION DES PERSONNES

La mise en place de ce programme d'éducation thérapeutique et le recrutement des patients auront lieu dans le service des urgences pédiatriques du CHU Robert Debré, 66 000 entrées par an sont dénombrées.

Les séances d'ETP se dérouleront immédiatement après la fin de la consultation par le médecin prescripteur, une fois l'accord de la famille obtenu. Elles auront lieu dans le même box de consultation. Les ordonnances remises au patient et/ou aux parents serviront de support à la séance.

Une information complète et loyale sera fournie au sujet, et une lettre d'information lui sera remise (Annexe 1) : elle précise les objectifs, les méthodes et la durée de la participation (pour un sujet donné) et elle rappelle que le patient peut à tout moment et sans préjudice fonctionnel, retirer son consentement.

Cette information sera notifiée dans le dossier médical du patient.

7. GESTION DES EVENEMENTS INDESIRABLES

A priori, il n'y a pas d'effets indésirables graves attendus en lien avec la recherche : les évènements indésirables graves attendus sont ceux des médicaments prescrits, et donc sans lien de causalité avec la recherche. On ne peut toutefois exclure une mauvaise compréhension des consignes fournies lors de la séance d'éducation.

8. GESTION DES DONNEES

8.1. Cahiers d'observation :

Annexe 2

8.2. Traitement des données et conservation des documents et des données

Toutes les informations requises par le protocole doivent être fournies dans le cahier d'observation.
Les données devront être transférées dans les cahiers d'observation au fur et à mesure qu'elles sont obtenues qu'il s'agisse de données cliniques ou para-cliniques. Les données devront être copiées de façon nette et lisible à l'encre noire dans ces cahiers (ceci afin de faciliter la duplication et la saisie informatique).
Les données erronées dépistées sur les cahiers d'observation seront clairement barrées et les nouvelles données seront copiées sur le cahier avec les initiales et la date par le membre de l'équipe de l'investigateur qui aura fait la correction.

Les données seront recueillies dans le cahier d'observation et seront anonymisées. Seuls les investigateurs auront accès aux données du patient et s'assureront de la confidentialité des informations.
Les données informatisées sur un fichier seront déclarées à la CNIL selon la procédure adaptée au cas.
Les documents spécifiques de la recherche seront archivés par l'investigateur après la fin de la recherche pendant 10 ans

9. ASPECTS STATISTIQUES

9.1. Justification de la taille de l'échantillon

Le calcul de l'échantillon a été réalisé sur le critère de jugement principal.
Pour une étude de supériorité, le pourcentage de satisfaction pour le critère principal dans le groupe contrôle est évalué à 65%. On escompte une amélioration de 15% soit 80% de satisfaction pour le critère principal dans le groupe ETP antibiotique. Le risque de première espèce est fixé à 5% et la puissance à 80%. Il faut donc au moins 136 sujets par groupe. Compte tenu d'une marge nécessaire on inclura 150 enfants par groupe soit 300 au total sur une période de 2 ans

9.2. Description des méthodes statistiques prévues y compris du calendrier des analyses intermédiaires prévues

Les analyses statistiques seront réalisées dans l'unité d'épidémiologie clinique de l'Hôpital Robert DEBRE, sous la responsabilité du Dr Corinne Alberti. Le logiciel statistique utilisé est SAS v 9.12.

Les variables qualitatives seront décrites sous forme d'effectifs et pourcentages, les variables quantitatives sous forme de médianes (quartiles) ou moyennes (déviation standard) en fonction de la distribution gaussienne ou non. Les comparaisons utiliseront des tests paramétriques ou non paramétriques en fonction de la nature et de la distribution des variables.
Les données obtenues à partir des échelles de Likert à 5 points (cf Echelles de Likert chapitre 5.5) seront dichotomisées. Les réponses 1, 2 et 3 sont considérées comme des réponses défavorables et les réponses 4 et 5 comme favorables pour les questions 2, 3, 4, 6, 7, 9 et 10. Les réponses 3, 4 et 5 sont considérées comme des réponses défavorables et les réponses 1 et 2 comme favorables pour les questions 5 et 8. [5]
Les données seront analysées selon le principe de l'intention de traiter, c'est-à-dire que les patients seront analysés selon leurs groupes de randomisation, et ce quelque soit la séance d'ETP effectivement reçue.
Le critère principal et les critères principaux seront analysés grâce au test du Chi-2.

10. ASPECTS ETHIQUES ET LEGAUX

10.1. Obligations légales (Rôle du gestionnaire, CPP, CCTIRS, CNIL)

Rôle de gestionnaire
Dr François Angoulvant est le gestionnaire de cette recherche conformément au 2° alinéa de l'article L.1121-1 du code de la Santé Publique. Il soumet le dossier à l'avis du Comité de Protection des Personnes CPP Ile De France n°2.
L'AP-HP via le DRCD a pris en charge le paiement de la taxe auprès de l'Agence Française de Sécurité Sanitaire des Produits de Santé.
Soumission au CPP
L'avis du comité mentionné ci-dessus est notifié dans la note d'information donnée aux personnes concernées.
Déclaration CNIL
Cette recherche est soumise à la loi du 6 janvier 1978 relative à l'informatique, aux fichiers et aux libertés. Modifiée par la loi n° 2004-801 de 6 août 2004.
Avant son début effectif, le traitement des données collectées dans la recherche est subordonnée à la saisine du Comité Consultatif sur le Traitement de l'Information en matière de Recherche dans la domaine de la Santé (CCTIRS) puis de la Commission Nationale de l'Informatique et des Libertés (CNIL). La recherche fera l'objet d'une déclaration unitaire.

Les informations relatives aux droits des personnes participant à cette recherche sont intégrées dans la note d'information

10.2. Modifications de la recherche

Toute modification substantielle sera soumise au CPP pour avis.

11. BIBLIOGRAPHIE

[1] Ammentorp J, Mainz J, Sabroe S. Parents' priorities and satisfaction with acute pediatric care. Archives of pediatrics & adolescent medicine. 2005 Feb;159(2):127-31.

[2] Hall JA, Roter DL, Katz NR. Meta-analysis of correlates of provider behavior in medical encounters. Med Care. 1988 Jul;26(7):657-75.

[3] Kardas P, Devine S, Golembesky A, Roberts C. A systematic review and meta-analysis of misuse of antibiotic therapies in the community. International journal of antimicrobial agents. 2005 Aug;26(2):106-13.

[4] Pechere JC. Patients' interviews and misuse of antibiotics. Clin Infect Dis. 2001 Sep 15;33 Suppl 3:S170-3.

[5] Pechere JC, Hughes D, Kardas P, Cornaglia G. Non-compliance with antibiotic therapy for acute community infections: a global survey. International journal of antimicrobial agents. 2007 Mar;29(3):245-53.

[6] Wu YP, Roberts MC. A meta-analysis of interventions to increase adherence to medication regimens for pediatric otitis media and streptococcal pharyngitis. Journal of pediatric psychology. 2008 Aug;33(7):789-96.

[7] Chappuy H, Treluyer JM, Gary A, Pons G, Cheron G. [Compliance with medication in children]. Arch Pediatr. 2005 Jun;12(6):921-3.

[8] Hoppe JE, Wahrenberger C. Compliance of pediatric patients with treatment involving antibiotic suspensions: a pilot study. Clin Ther. 1999 Jul;21(7):1193-201.

[9] Steele RW, Thomas MP, Begue RE. Compliance issues related to the selection of antibiotic suspensions for children. Pediatr Infect Dis J. 2001 Jan;20(1):1-5.

[10] Huang SS, Rifas-Shiman SL, Kleinman K, Kotch J, Schiff N, Stille CJ, et al. Parental knowledge about antibiotic use: results of a cluster-randomized, multicommunity intervention. Pediatrics. 2007 Apr;119(4):698-706.

[11] Colcher IS, Bass JW. Penicillin treatment of streptococcal pharyngitis. A comparison of schedules and the role of specific counseling. Jama. 1972 Nov 6;222(6):657-9.

[12] D'Ivernois JF, Gagnayre R. Apprendre à éduquer le patient : approche pédagogique 2e éd. Maloine ed. Paris 2004.

[13] Tubiana-Rufi N. L'enfant, la maladie, l'éducation thérapeutique ; point de vue Clinique. Le journal des professionnels de l'enfance 2004 (mars-avril):32-5.

[14] Antibiothérapie par voie générale en pratique courante: Recommandations de bonnes pratiques. AFSSAPS 2005.

[15] Schrag SJ, Pena C, Fernandez J, Sanchez J, Gomez V, Perez E, et al. Effect of short-course, high-dose amoxicillin therapy on resistant pneumococcal carriage: a randomized trial. Jama. 2001 Jul 4;286(1):49-56.

[16] Kajioka EH, Itoman EM, Li ML, Taira DA, Li GG, Yamamoto LG. Pediatric prescription pick-up rates after ED visits. Am J Emerg Med. 2005 Jul;23(4):454-8.

[17] Matsui D, Joubert GI, Dykxhoorn S, Rieder MJ. Compliance with prescription filling in the pediatric emergency department. Archives of pediatrics & adolescent medicine. 2000 Feb;154(2):195-8.

[18] Christakis DA, Wright JA, Taylor JA, Zimmerman FJ. Association between parental satisfaction and antibiotic prescription for children with cough and cold symptoms. Pediatr Infect Dis J. 2005 Sep;24(9):774-7.

[19] Mangione-Smith R, McGlynn EA, Elliott MN, McDonald L, Franz CE, Kravitz RL. Parent expectations for antibiotics, physician-parent communication, and satisfaction. Archives of pediatrics & adolescent medicine. 2001 Jul;155(7):800-6.

[20] Kardas P, Pechere JC, Hughes DA, Cornaglia G. A global survey of antibiotic leftovers in the outpatient setting. International journal of antimicrobial agents. 2007 Dec;30(6):530-6.

[21] McDonald HP, Garg AX, Haynes RB. Interventions to enhance patient adherence to medication prescriptions: scientific review. Jama. 2002 Dec 11;288(22):2868-79.

12. ANNEXES

ANNEXE 1 Note d’information

ANNEXE 2 Cahier d’observation

ANNEXE 3 liste des investigateurs et collaborateurs
